# The long-noncoding RNA MALAT1 regulates TGF-β/Smad signaling through formation of a lncRNA-protein complex with Smads, SETD2 and PPM1A in hepatic cells

**DOI:** 10.1371/journal.pone.0228160

**Published:** 2020-01-29

**Authors:** Jinqiang Zhang, Chang Han, Kyoungsub Song, Weina Chen, Nathan Ungerleider, Lu Yao, Wenbo Ma, Tong Wu

**Affiliations:** Department of Pathology and Laboratory Medicine, Tulane, Wenbo University School of Medicine, New Orleans, Louisiana, United States of America; Hungarian Academy of Sciences, HUNGARY

## Abstract

Recent studies have demonstrated the implication of long noncoding RNAs (lncRNAs) in a variety of physiological and pathological processes. However, the majority of lncRNAs are functionally unknown. The current study describes that the lncRNA MALAT1 regulates TGF-β/Smad signaling pathway through formation of a lncRNA-protein complex containing Smads, SETD2 and PPM1A. Our data show that this lncRNA-proteins complex facilitates the dephosphorylation of pSmad2/3 by providing the interaction niche for pSmad2/3 and their specific phosphatase PPM1A, thus terminating TGF-β/Smad signaling in hepatic cells. Based on these mechanistic studies, we performed further experiments to determine whether depletion of MALAT1 would augment cellular TGF-β/Smad signaling. We observed that MALAT1 depletion enhanced TGF-β/Smad signaling response, as reflect by amplification of Smad-mediated differentiation of induced pluripotent stem (iPS) cells to hepatocytes. Our experimental results demonstrate an important role of MALAT1 for regulation of TGF-β/Smad signaling in hepatic cells. Given the diverse functions of TGF-β/Smad pathway in various physiological and pathogenic processes, our results described in the current study will have broad implications for further understanding the role of MALAT1 in TGF-β/Smad pathway in human biology and disease.

## Introduction

High-throughput studies have indicated the fascinating complexity of the human transcriptome including abundant RNAs with no protein coding capacity[1–4]. The noncoding transcripts ranging in size from 200nt to longer than 100kb are assigned arbitrarily as the long noncoding RNAs (lncRNAs), which is the largest and most complex class of noncoding RNAs[3, 5]. The vast majority of lncRNAs are functionally unknown; only dozens of them have been described with biological roles, mainly through four archetypes of molecular mechanisms–acting as signals, as decoys, as guides, or as scaffolds[6]. Intriguingly, in each archetype, lncRNAs form protein-lncRNA complexes with some key protein factors to execute their functions[6, 7]. Therefore, there is a noticeable need to further dissect whether key protein factors of pivotal signaling pathways may form protein-lncRNA complexes, and whether these complexes may in turn affect the activity of their respective signaling pathways.

Smad transcription factors lie at the core of the transforming growth factor-β (TGF-β) pathway, which controls a plethora of cellular responses including development, stem cell maturation, and carcinogenesis, among others[8]. Smad protein factors, together with co-activators or co-inhibitors can bind to specific DNA sequences in promoter regions and regulate transcription activity of certain genes[9]. A recent study showed that Smad proteins could also bind to some primary microRNA transcripts and regulate their maturation[10]. Thus, we postulate that Smad proteins may form RNA-protein complexes with certain lncRNA molecules and these complexes may modulate the functions of Smads or related lncRNAs. To test this hypothesis, we carried out a series of RNA immunoprecipitation experiments using phospho-Smad2/3 antibodies in hepatic cells and observed that the lncRNA MALAT1 (metastasis-associated lung adenocarcinoma transcript 1) specifically binds to phospho-Smad2/3.

The lncRNA MALAT1, also known as NEAT2 (nuclear-enriched abundant transcript 2), is a highly conserved nuclear noncoding RNA among mammalians with length of more than 8 kb in human (which is localized exclusively in nuclear speckles) [11, 12]. Studies have shown that MALAT1 plays important roles in multiple cellular processes and diseases[13–18]. In the present study we describe a novel mechanism for MALAT1 interaction with phospho-Smad2/3, SETD2 and PPM1A in hepatic cells. Our data show that this MALAT1-protein complex facilitates the dephosphorylation of pSmad2/3 by providing the interaction niche for pSmad2/3 and their specific phosphatase PPM1A, thus terminating TGF-β/Smad signaling in hepatic cells. Our experimental results disclose a novel mechanism by which MALAT1 negative regulates cellular TGF-β/Smad signaling.

## Materials and methods

### Materials

Specific antibodies were purchased from the following commercial sources: Anti-AFP, anti-ALB, anti-CD44, anti-Evi1, anti-flag (mouse), anti-HA, anti-HNF4α, anti-H3, anti-H3K36me3, anti-Myc, anti-OCT4A, anti-P300, anti-PPM1A (rabbit), anti-pSmad2 (S465/467), anti-pSmad2 (S245/250/255), anti-pSmad3 (s423/425), anti-Smad2, anti-Smad3 (rabbit), anti-SnoN, anti-Sox2, anti-TAT, and normal rabbit IgG from Cell Signaling Technology (Danvers, MA); anti-PPM1A (mouse), anti-SC35, and anti-SETD2 were from Abcam (Cambridge, MA); Anti-Smad4 and normal mouse IgG were from Santa Cruz Biotechnology (Santa Cruz, CA); Anti-β-actin, and anti-flag (rabbit) from Sigma-Aldrich (St. Louis, MO); Alexa594 goat anti-mouse IgG from Life Technology (Carlsbad, CA); Dylight488 goat anti-rabbit IgG from Vector Labs (Burlingame, CA).

### Cell culture

Human transformed hepatocytes (Hep3B, SK-Hep1, PLC/PRF/5, and Huh7) were cultured in Dulbecco's Modified Eagle Medium (DMEM) with 10% heat-inactivated fetal bovine serum. The immortalized human hepatocytes (THLE2) were maintained in complete BEGM^TM^ Medium (Lonza, Allendale, NJ) supplemented with 10% heat-inactivated fetal bovine serum. All cells were cultured at 37ºC in a humidified 5% CO2 incubator.

### RNA immunoprecipitation

Cells cultured in 100-mm dishes were fixed by 1% paraformaldehyde for 10 minutes and quenched by 125mM Glycine; the cells were then collected and washed twice with ice-cold phosphate buffered saline containing protease inhibitor cocktail and phosphatase inhibitor cocktail (Roche, Mannheim, Germany). After that, cell pellet was re-suspended in 200ul Buffer A (5mM PIPES pH8.0, 85mM potassium chloride, 0.5% NP-40, protease inhibitor cocktail, phosphatase inhibitor cocktail and RNase inhibitor) and placed on ice for 10 minutes. The crude nuclei fraction was pelleted and washed once in buffer A (without NP-40), then re-suspended in 500ul of Buffer B (10mM EDTA, 50mM Tris-HCl pH8.1, protease inhibitor cocktail, phosphatase inhibitor cocktail and RNase inhibitor) and placed on ice for 10 minutes. After centrifuged at 14000 rpm for 10 minutes at 4 ºC, the supernatant was diluted 10-fold into IP Buffer (0.01% SDS, 1.1% Triton X-100, 1.2mM EDTA, 16.7mM Tris pH8.1, 167mM sodium chloride, protease inhibitor cocktail, phosphatase inhibitor cocktail and RNase inhibitor). One microgram of indicated antibody was added to 1ml diluted supernatant; the samples were rotated slowly at 4 ºC overnight. Subsequently, 50μl of Protein A/G Agarose beads (Santa Cruz) was added to each tube and the samples continued to be rotated for 2 hours. Immune complex was pelleted and washed successively by Low-slat Buffer (0.1% SDS, 1% Triton X-100, 2mM EDTA, 20mM Tris-HCl pH 8.1, 150mM NaCl), High-salt Buffer (0.1% SDS, 1% Triton X-100, 2mM EDTA, 20mM Tris-HCl pH 8.1, 500mM NaCl), LiCl Buffer (0.25M LiCl, 1% NP40, 1% deoxycholate, 1mM EDTA, 10mM Tris-HCl pH 8.1), and Tris-EDTA(pH8.0) Buffer. 250μl fresh prepared Elution Buffer (1% SDS, 0.1M NaHCO3, nuclease inhibitor) was added to the complexes and the samples were incubated for 15 minutes with rotating to elute the complex; supernatant was collected by centrifugation at 8000 rpm, 2 minutes. Elution was repeated and elute combined for a total of 500μl. 3M NaCl was added to elute to a final concentration of 200mM and the samples were placed at 65ºC for at least 2 hours to reverse crosslinking. Next, 20μl of 1M Tris-Cl pH 6.5, 10μl of 0.5M EDTA, and 20μg of Proteinase K was added and the samples were incubated at 42ºC for 45 minutes. After incubation, the samples were subjected to phenol:chloroform:isoamyl alcohol extraction and ethanol precipitation with Glycoblue (Ambion, Carlsbad, CA) as a carrier. Pellets were washed once in 75% ethanol, air-dried briefly, and re-suspended in 20μl of DEPC-treated water. Then the RNA (treated with DNAse I to remove DNA) was ready for reverse transcription and quantitative PCR analysis.

### Induced pluripotent stem (iPS) cell differentiation

Human iPS cells (ACS-1011) were obtained from ATCC (Manassas, VA) and maintained with the stem cell culture medium SFM XF/FF (ACS-3002). The three step protocol of hepatocyte-like cells induction from iPS cells was modified on the base of previous publications [19]. First, 70% confluent iPS cells were cultured with Roswell Park Memorial Institute (RPMI)/B27 medium containing 100ng/mL activin A or 10ng/ml TGF-β1, along with 50ng/mL Wnt3a, and 10ng/mL HGF (R&D Systems) for 3 days of endodermal induction. Then, RPMI/B27 was replaced by hepatic commitment medium (DMEM containing 20% knockout serum replacement, 1mM L-glutamine, 1% nonessential amino acids, 0.1mM 2-mercaptoethanol, and 1% dimethyl sulfoxide) for another 4 days. Finally, the cells were incubated in Iscove's modified Dulbecco's medium (IMDM) supplemented with 20ng/mL oncostatin M (Invitrogen), 0.5μM dexamethasone, and 50 mg/mL ITS premix (BD Biosciences, San Jose, CA) for 5 days to induce hepatocyte maturation.

### Statistical analysis

Data were presented as mean ± standard deviation from a minimum of three replicates as indicated in the figure legends. Difference between groups was evaluated by SPSS 19.0 statistical software with one-way analysis of variance, Student’s t test, Mann–Whitney U test or Fisher exact test. A p-value <0.05 was considered as statistically significant.

Additional methods, including plasmids and siRNA transfection, cell proliferation WST1 assay, luciferase activity assay, quantitative real-time PCR, immunoprecipitation, DNA pull down, Western blot, fluorescent in situ hybridization (FISH), immunofluorescence, and hepatic spheroid formation assay, as well as a list of primers used in RT-PCR analysis are described in S1 File.

## Results

### TGF-β activated Smad2/3 associate with MALAT1

**Fig 1:**
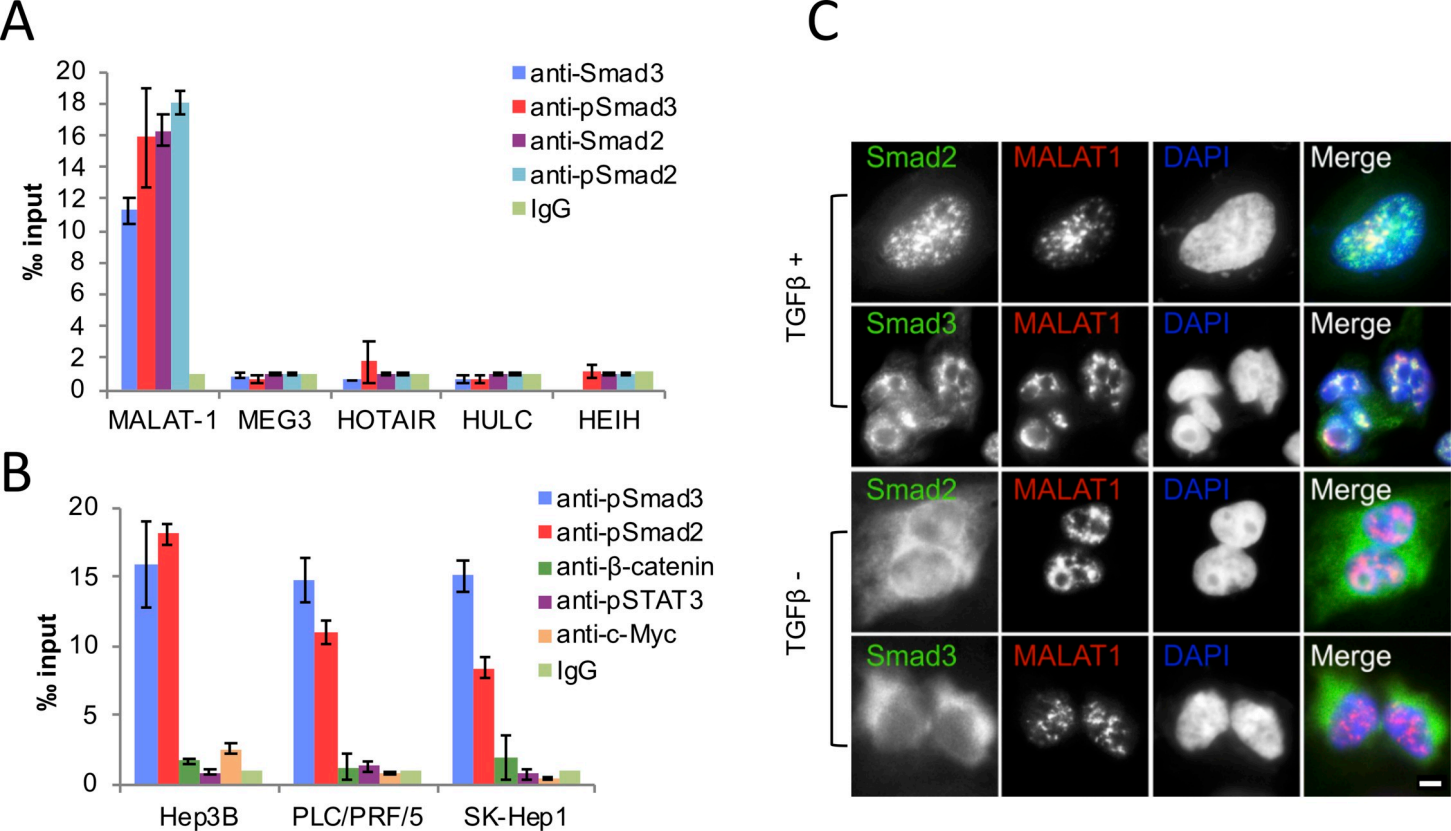
MALAT1 binds to pSmad2/3 specifically. **A,** RNA immunoprecipitation assays of multiple lncRNAs with antibodies targeting Smad2, Smad3, pSmad2 or pSmad3 in Hep3B treated with TGF-β1 for 1 hour. The precipitated lncRNAs were assessed by qRT-PCR. **B,** RNA immunoprecipitation assays of MALAT1 with indicated antibodies in Hep3B, PLC/PRF/5 and SK-Hep1 cells treated with TGF-β1 for 1 hour. The precipitated lncRNA was assessed by qRT-PCR. **C,** Co-localization of pSmad2 or pSmad3 with MALAT1 in the nuclei of Hep3B cells as demonstrated by FISH in combination with IF staining (scale bar 10 μm). All data are shown as means ± SD (n = 3).

To identify lncRNAs that might be associated with phospho-Smad2/3, we carried out a small-scale screening by employing RNA immunoprecipitation experiments in human transformed hepatocytes (PLC/PRF/5, SK-Hep1, and Hep3B) treated with TGF-β. We observed that MALAT1 was enriched specifically by antibodies targeting total or phospho-Smad2/3 (Fig 1A). To further verify the association specificity between pSmad2/3 and MALAT1, we used several other nuclear proteins antibodies, such as anti-pSTAT3, anti-β-catenin, and anti-c-Myc, as control of the RNA immunoprecipitation experiments; the results show that MALAT1 associates exclusively with Smad2/3 (Fig 1B). We next utilized immunofluorescence combined with FISH staining to determine whether TGF-β1 was able to induce pSmad2/3 association with MALAT1 (hence co-localization). We observed that phosphorylated Smad2/3 entered into the nucleus, accumulated and overlapped with MALAT1 in TGF-β1 treated Hep3B cells (Fig 1C). Our further FISH-IF analysis indicated that MALTA1 was co-localized with the nuclear speckle marker SC-35 (Fig S1A in S2 File). Meanwhile, TGF-β1 did not influence MALAT1 nuclear localization and transcription as indicated by FISH-IF staining and RT-PCR analysis, respectively (Fig 1C and Fig S1 in S2 File).

### SETD2 mediates pSmad2/3 association with MALAT1

**Fig 2:**
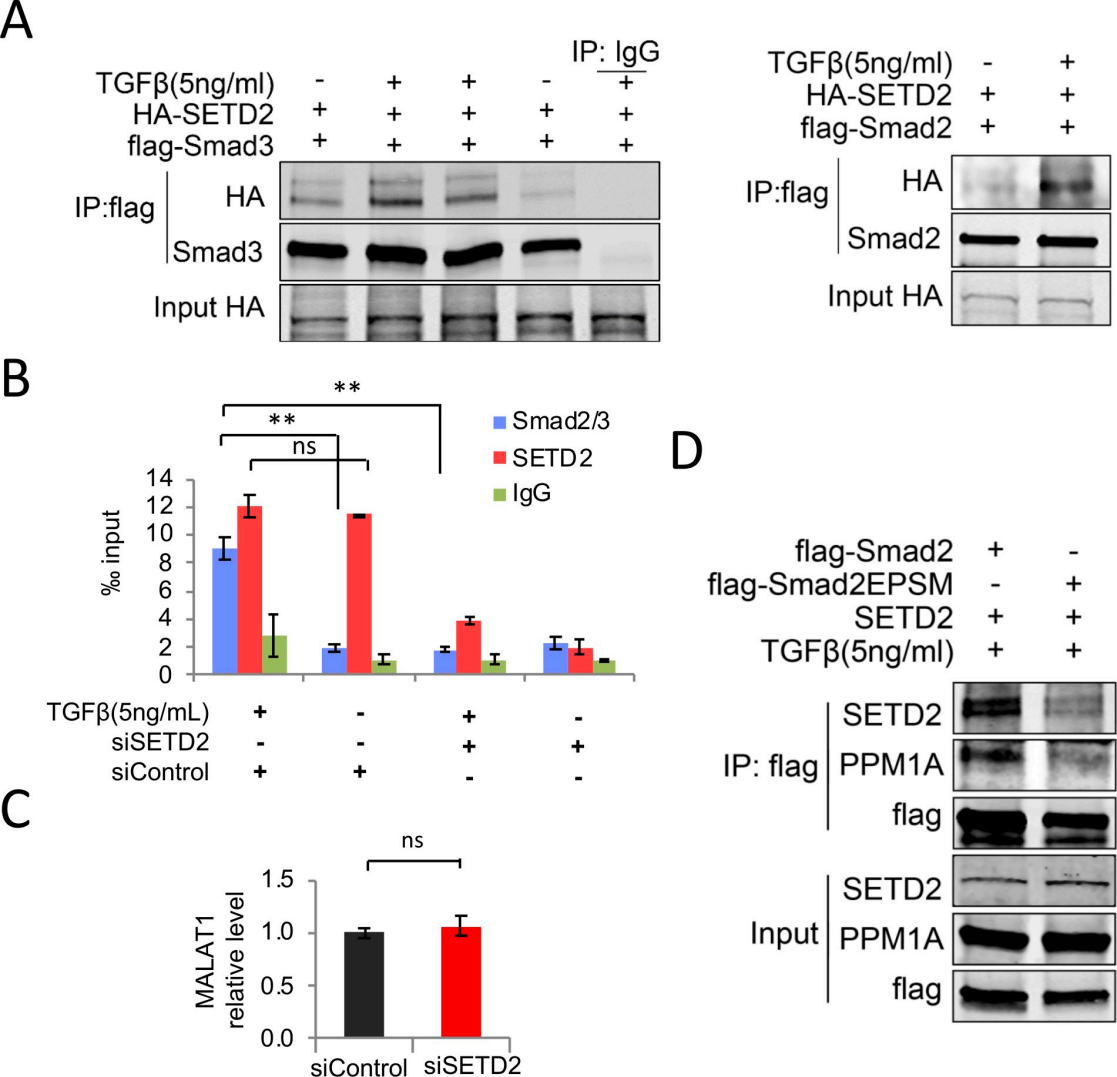
SETD2 mediates the binding of MALAT1 and pSmads. **A,** Smad2 and Smad3 bind to SETD2 protein. Hep3B cells co-transfected with HA-SETD2 and flag-Smad2/3 expression plasmids were treated with TGF-β1 for 1 hour. Cells were harvested and subjected to immunoprecipitation assays. **B,** RNA immunoprecipitation of MALAT1 by using anti-Smad2/3 or anti-SETD2 antibodies in Hep3B cells (with siSETD2 or siControl transfection) treated with 5ng/mL TGF-β1 or vehicle for 2 hours. The precipitated MALAT1 was assessed by qRT-PCR. **C.** qRT-PCR results indicated that SETD2 knockdown did not affect MALAT1 level in Hep3B cells. The data are presented as means ± SD (n = 3; **p < 0.01; ns, no statistical significance). **D,** Smad2 linker region mutations decrease its capability to form protein complex with PPM1A and SETD2 proteins. PLC/PRF/5 cells co-transfected with SETD2 and flag-Smad2 or flag-Smad2EPSM (T220V, S245A, S250A and S255A, Erk/Pro-directed kinase Site Mutated Smad2) expression plasmids were incubated with TGF-β1 for 1 hour. The treated cells were then harvested and subjected to immunoprecipitation and immunoblotting with indicated antibodies.

The next question we asked is how MALAT1 interacted with pSmad2/3, i.e., through direct binding or via other molecules indirectly. Yang et al[14] had identified numerous MALAT1-binding proteins by using RNA pull-down combined with mass spectrum analysis; it came to our attention that SET domain containing 2 (SETD2) (also known as Huntington-interacting protein B) was identified as one of the MALAT1 binding proteins in their results. In another study, by using yeast two-hybrid and co-immunoprecipitation methods, Wang et al[20] reported that SETD2 protein could form complex with phospho-Smad in the liver; this phenomenon was confirmed in our study by using Hep3B cells (Fig 2A). On the basis of these results, we reasoned that SETD2 might mediate the interaction between MALAT1 and phospho-Smad. To validate this hypothesis, we performed RNA immunoprecipitation experiments in Hep3B cells with or without SETD2 depletion by siRNA (successful depletion of SETD2 was verified by Western blotting, as shown in Fig S2 in S2 File). RNA-IP and qRT-PCR assays showed that SETD2 depletion significantly decreased Smad2/3 association with MALAT (Fig 2B). Unlike Smad2/3 association with MALAT1, we observed that SETD2 association with MALAT1 was not influenced by TGF-β treatment (Fig 2B). In addition, our data showed that SETD2 depletion did not influence MALAT1 expression (Fig 2C). These results demonstrate that MALAT1 interaction with pSmad2/3 is mediated by SETD2.

SETD2 belongs to H3K36-specific methyltransferase family. It consists of a catalytic SET domain, a C-terminal RNA polymerase II interaction domain, and a WW domain for binding to target proteins, such as Proline-Rich PY (Pro-Pro-X-Tyr) motif[21]. There are four threonine/serine residues (T220, S245, S250, S255) which are localized around a PY motif in the Smad2 linker region; phosphorylation of these four threonine/serine residues by CDK8/9 is required for the binding of PY motif to WW domain[22–24]. To determine whether PY-WW motif interaction involve in the binding of phosphorylated Smad2/3 to SETD2, PLC/PRF/5 cells were transfected with plasmids expressing linker region mutated Smad2 (Smad2-EPSM, T220V, S245A, S250A, S255A)[23] and then subjected to immunoprecipitation assays. We observed that mutations of the linker threonine/serine residues abolished Smad2 binding to SETD2 (Fig 2D), despite that mutated Smad2-EPSM can be phosphorylated at the C-terminal SSXS motif and translocated to nuclei as wild type Smad2 upon TGF-β treatment (Fig S3 in S2 File). These findings suggest the essential roles of Smad2 PY motif and SETD2 WW domain in the interaction of these two proteins.

### Depletion of MALAT1 or SETD2 enhances Smad2/3 signaling

**Fig 3:**
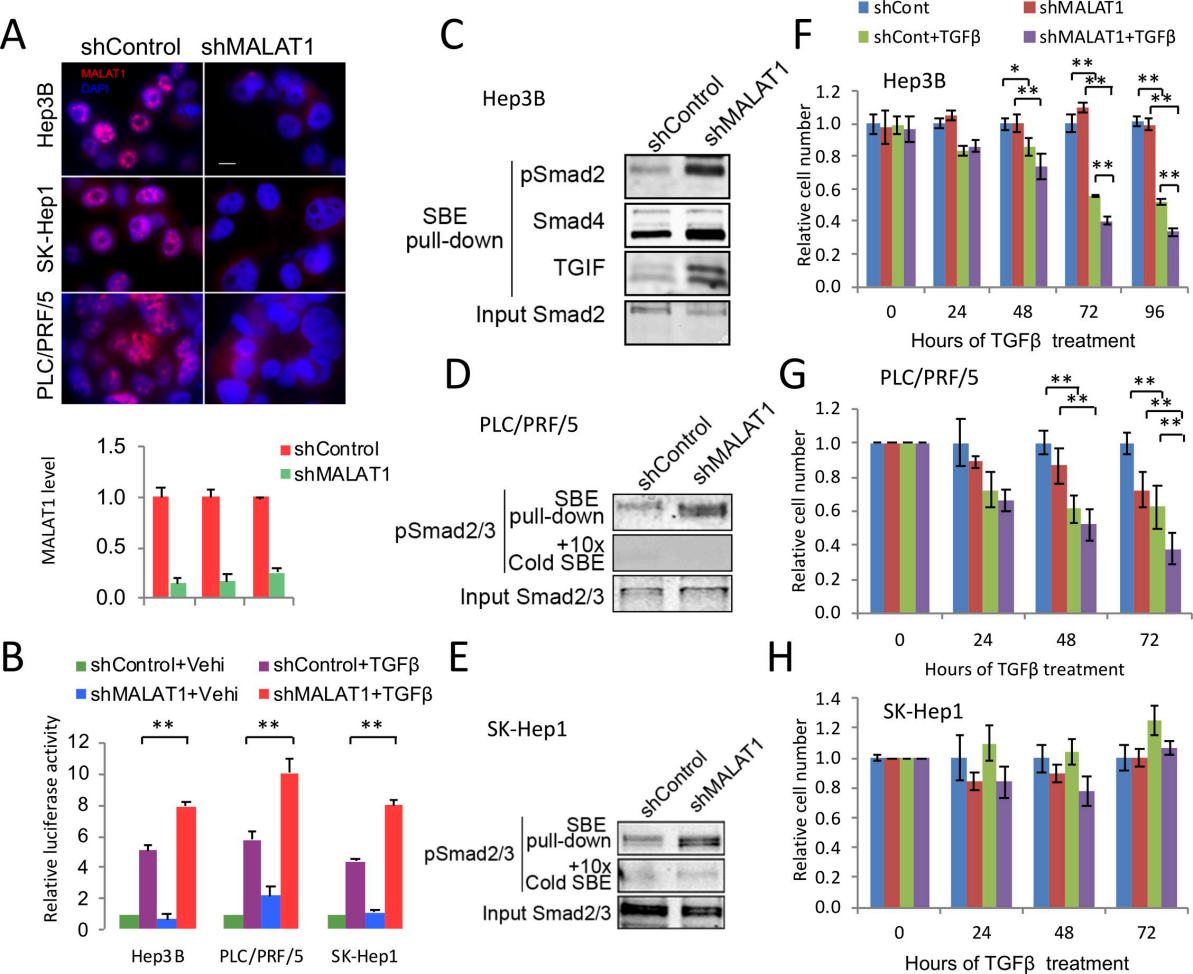
Depletion of MALAT1 enhances the effect of TGF-β. **A,** The levels of MALAT1 were assessed by FISH (upper) and qRT-PCR (lower) in Hep3B, SK-Hep1 and PLC/PRF/5 cells stably transfected with pGFP-V-RS-shMALAT1 or scramble control plasmid (scale bar 10 μm). **B,** Smad reporter activity in Hep3B, SK-Hep1 and PLC/PRF/5 cells with or without MALAT1 depletion. Twenty-four hours after transfection with p3TP-lux reporter plasmid, MALAT1 depleted and control cells were stimulated with 5ng/ml TGF-β1 or vehicle for additional 24 hours. The cell lysates were obtained for dual-luciferase activity assay. The data are presented as means ± SD (n = 3; *p < 0.05, **p < 0.01). **C-E,** DNA pull-down assay. Equal amount of cell lysates from MALAT1 depleted or control Hep3B (**C**), PLC/PRF/5 (**D**) or SK-Hep1 (**E**) cells stimulated with TGF-β1 (5 ng/ml for 2 hours) were pulled down with biotinylated SBE (Smads binding elements) DNA probe[40, 41], which were followed by immunoblotting with indicated antibodies. **F-H,** Cell proliferation assay. Equal numbers of MALAT1 depleted or control Hep3B (**F**), PLC/PRF/5 (**G**) or SK-Hep1 (**H**) cells were seeded into 96-well plates. The cells were treated with TGF-β1 (1 ng/ml) or vehicle control for 0–96 hours and cell proliferation was measured by WST-1 at indicated time points (n = 6). Statistical analysis was performed by using one-way ANOVA method and the data are presented as means ± SD (* P < 0.05; ** P < 0.01).

To determine the functional impact of MALAT1 on TGF-β signaling, we utilized shRNA to knockdown MALAT1. The efficiency of MALAT1 knockdown was confirmed by FISH and qRT-PCR analyses (Fig 3A**)**. Our data showed that MALAT1 knockdown increased Smad2/3 activities as determined by p3TP-lux luciferase reporter activity assay (Fig 3B). This result was further supported by the observation that depletion of MALAT1 by 2’-OME-gamper ASO also increased the p3TP-lux luciferase reporter activity (Fig S4A in S2 File). By using DNA pull-down assay, we observed that MALAT1 knockdown increased the binding of pSmad2/3 to the Smad-binding element (SBE) (Fig 3C–3E). These results indicate that MALAT1 knockdown enhances the transcription activity of pSamd2/3. Consistent with the fact that Smad4 and TGIF (TGF-β induced factor) are components of activated Smad2/3 complex[25], we also detected increased Smad4 and TGIF in pSmad2/3-SBE complex in MALAT1 knockdown cells. Given that SETD2 protein mediates Smad2/3 association with MALAT1 (as documented above), we anticipated that knockdown of SETD2 would undermine Smad2/3 interaction with MALAT1. Indeed, we observed that knockdown of SETD2 by siRNA increased both p3TP-lux luciferase reporter activity and the binding of pSmad2/3 to SBE in Hep3B cells with TGF-β1 stimulation; conversely, forced overexpression of SETD2 partially inhibited Smads pro-transcription activity (Fig S4B and S4C in S2 File).

TGF-β induces cytostasis in PLC/PRF/5 and Hep3B cells, but not in SK-Hep1 cells[26, 27]. In accordance with this phenomenon, MALAT1 knockdown enhanced the growth inhibitory effect of TGF-β1 in Hep3B and PLC/PRF/5 cells (Fig 3F and 3G), but not in SK-Hep1 cells (Fig 3H). Histone H3K36 trimethylation (H3K36Me3), catalyzed by SETD2[21], is a transcriptional mark and has important implications in TGF-β-mediated EMT process[28]. To assess whether MALAT1 might affect H3K36Me3 status through interaction with SETD2 and thus affect cell response to TGF-β, we measured the levels of HK36Me3 in MALAT1 knockdown and control cells with or without TGF-β treatment. While TGF-β1 treatment slightly increased HK36Me3 level in both MALAT1 knockdown and control cells (Fig S5A in S2 File), we observed that MALAT1 depletion did not significantly alter the status of H3K36Me3 (Fig S5B in S2 File).

### MALAT1 and SETD2 are implicated in pSmad dephosphorylation

**Fig 4:**
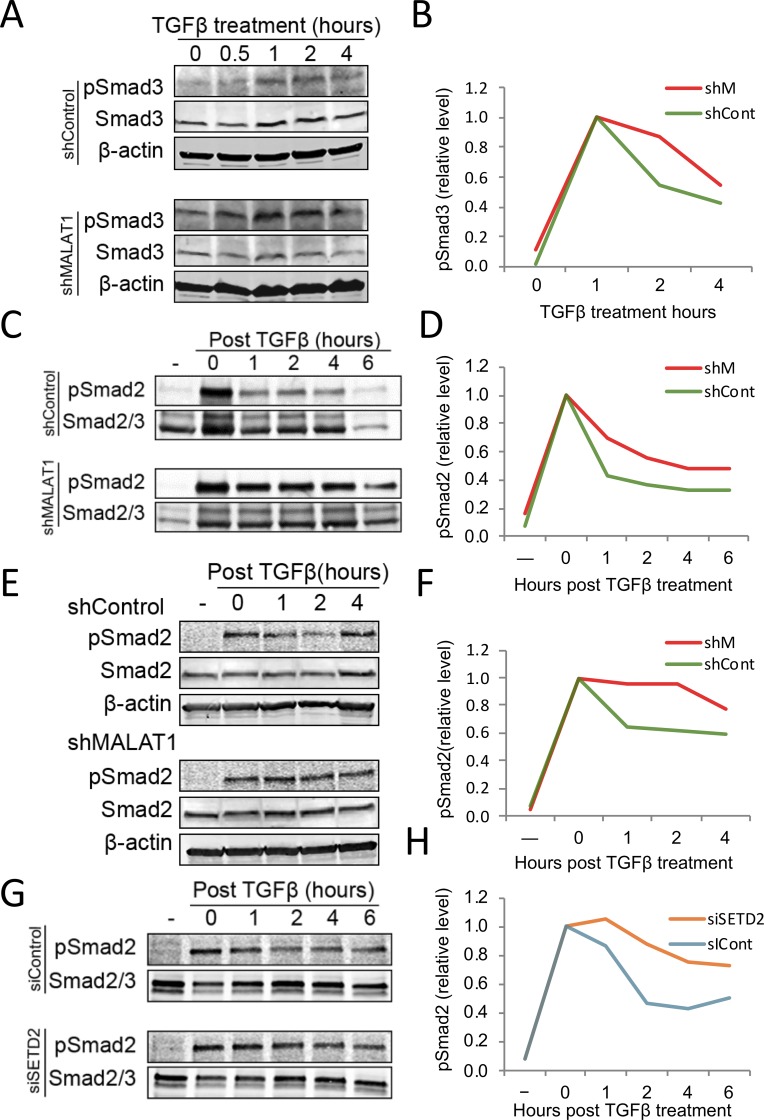
Depletion of MALAT1 reduces Smad2/3 dephosphorylation. **A, B,** Time course of Smad3 phosphorylation. MALAT1 depleted or control Hep3B cells were treated with 5 ng/ml of TGF-β1 for the indicated time periods. Phospho-Smad3 and total Smad3 were analyzed by Western blotting. Line graph demonstrates relative intensity ratio of phospho-Smad3 to total Smad3, the relative level of phospho-Smad3 at 1-hour time point was set as 1. **C-H,** Time course of Smad2 dephosphorylation in cells post TGF-β1 treatment. MALAT1 depleted or control Hep3B cells **(C, D),** MALAT1 depleted or control PLC/PRF/5 cells **(E, F)** and SETD2 knockdown and control Hep3B cells (**G, H**) were treated with 5 ng/ml of TGF-β1 for 1 hour, followed by TGF-β1 washout and simultaneous addition of 10 μM TGF-βR Inhibitor SB431542 and 10 μM proteasome inhibitor MG-132. The line graphs (**D**, **F** and **H**) show relative ratio of pSmad2 level over total Smad2 (the value at time point 0 was set as 1). The band intensities from representative Western blot results were quantified by Odyssey software (LI-COR Bio, Lincoln NE).

To investigate the mechanism underlying MALAT1 effect on TGF-β signaling, we examined the levels of several Smad co-activators or co-inhibitors in MALAT1 knockdown and control cells. The expression levels of these Smad co-factors did not significantly differ in cells with or without MALAT1 depletion (Fig S6 in S2 File); conversely, the level of phospho-Smad was increased in MALAT1 knockdown cells compared to the control cells. We next utilized nuclear fractionation and western blotting analysis to determine the levels of pSmad2/3 in the nuclear pool of hepatic cells with or without MALAT1 knockdown. Our data showed that TGF-β treatment induces the phosphorylation of Smad2/3 in the nucleus and that the levels of pSmad2/3 in the nuclear pool of hepatic cells with MALAT1 knockdown were higher than that in cells without MALAT1 knockdown (Fig S7 in S2 File). Enhanced phospho-Smad in MALAT1 knockdown cells was also confirmed by time course experiments (Fig 4A and 4B). The dynamic change in pSmad over time in post TGF-β-treated cells furthers support that the higher levels of pSmad2/3 in MALAT1 depleted cells are due to slower dephosphorylation rate (Fig 4C–4F). In consistence with p3TP-lux luciferase and SBE pull-down results, we also observed that SETD2 knockdown prevented pSmad2 dephosphorylation (Fig 4G and 4H). Collectively, the above results demonstrate that depleting MALAT1 or disassociating the MALAT1-pSmad2/3 complex (via SETD2 knockdown) amplifies cell response to TGF-β1 by preventing pSmad2/3 dephosphorylation.

### MALAT1 facilitates SETD2-pSmad2/3-PPM1A complex formation

**Fig 5:**
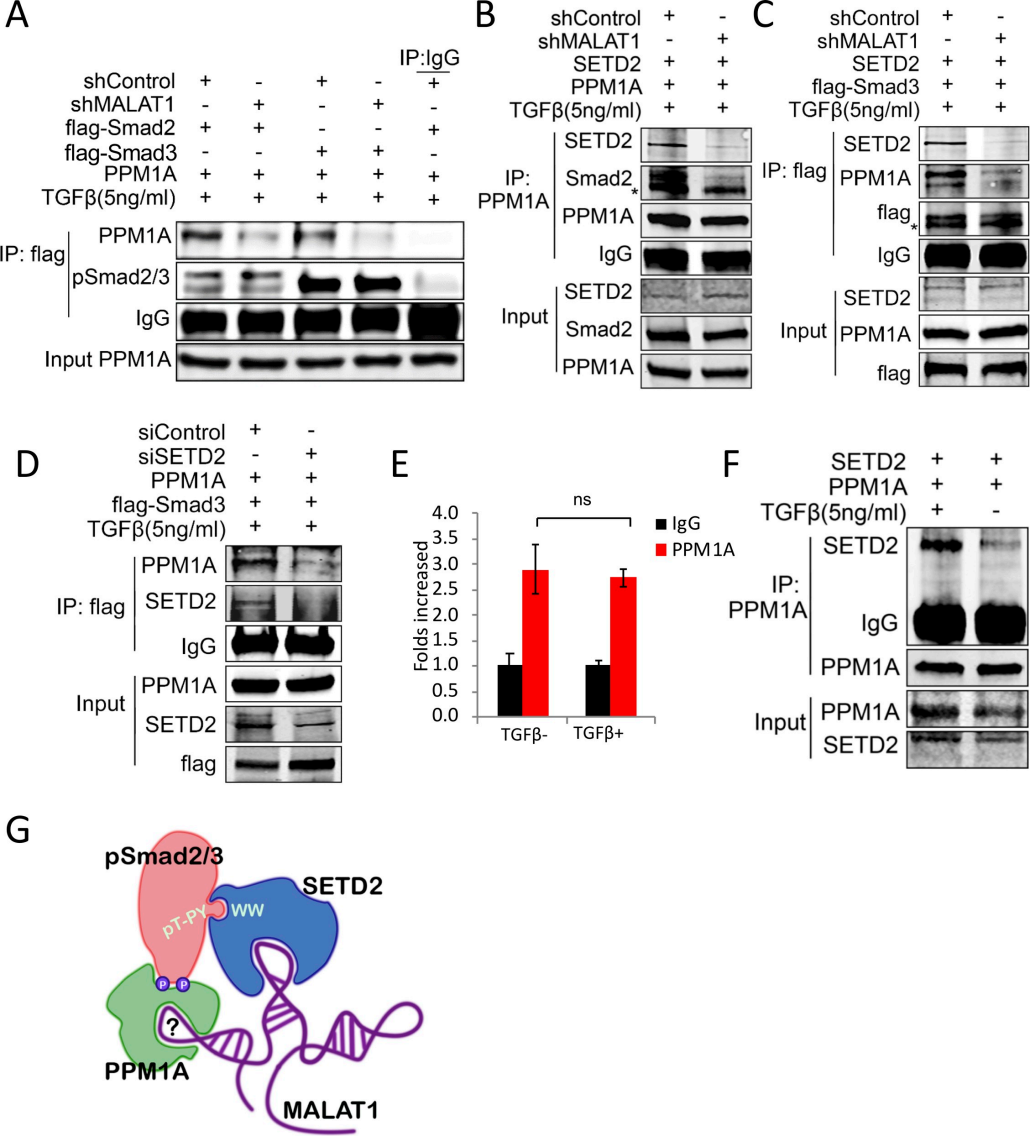
MALAT1 facilitates the SETD2-pSmad2/3-PPM1A complex formation. **A-C,** MALAT1 depletion prevents SETD2-pSmad2/3-PPM1A complex formation. MALAT1 depleted or control Hep3B **(A)** or PLC/PRF/5 **(B, C)** cells were transfected with flag-Smad2/3, PPM1A or SETD2 expression plasmids (as indicated at the top of each panel) and the cells were treated with TGF-β1 for 1–2 hours. The cell lysates were then subjected to IP and Western blotting analyses using indicated antibodies. **D,** SETD2 depletion prevents pSmad2/3 binding to PPM1A. Cell lysates from SETD2-depleted and control PLC/PRF/5 cells (co-transfected with PPM1A and flag-Smad3 expression plasmids) were subjected to co-immunoprecipitation using anti-flag antibody followed by Western blotting for SETD2 and PPM1A. **E,** RNA immunoprecipitation of MALAT1 by using anti-PPM1A antibodies in Hep3B cells with or without TGF-β1 treatment (5ng/ml for 1 hour); precipitated MALAT1 was assessed by qRT-PCR. The data are shown as mean ± SD (n = 3; ns, no statistical significance). **F,** Co-immunoprecipitation of PPM1A and SETD2 in PLC/PRF/5 cells (co-transfected with SETD2 and PPM1A expression plasmids) with or without TGF-β1 treatment. **G,** A diagram illustrating that MALAT1 acts as a scaffold to facilitate the SETD2-pSmad2/3-PPM1A protein complex formation. This complex facilitates the de-phosphorylation of phorspho-Smad2/3 and thus the termination of Smad2/3 signaling. *: non-specific bands.

PPM1A is known to bind phospho-Smad2/3 and function as a pSmad-phosphatase to terminate TGF-β signaling[29]. To investigate the possible role of MALAT1 and SETD2 in the interaction between PPM1A and phospho-Smad2/3, we performed immunoprecipitation in MALAT1 knockdown and control cells transfected with plasmids expressing PPM1A and flag-Smad2 or flag-Smad3. We observed that MALAT1 knockdown noticeably undermined the binding between PPM1A and phospho-Smad2/3 (Fig 5A) and reduced the interactions among phospho-Samd2/3, PPM1A and SETD2 (Fig 5B and 5C). Consistent with the notion that SETD2 acts as a molecular bridge between MALAT1 and pSmad2/3, knockdown SETD2 with siRNA considerably prevented the interaction between phospho-Smads and PPM1A (Fig 5D). The role of SETD2 and MALAT1 as connecting molecules to link PPM1A and pSmads was further supported by the evidence that four linker threonine/serine residues mutation (which demolishes the interaction between SETD2 and pSmad2) significantly reduced pSmad2 binding to PPM1A (Fig 2D). Additional RNA and protein immunoprecipitation assays show that PPM1A binding to MALAT1 is independent of TGF-β treatment (Fig 5E), whereas TGF-β treatment is required for PPM1A binding to SETD2 (Fig 5F).

The above results indicate that MALAT1, together with SETD2, forms a scaffold that facilitates the interaction between pSmad2/3 and PPM1A; the assembled lncRNA-proteins apparatus leads to the dephosphorylation of pSmad2/3 and thus termination of TGF-β/Smads signaling (illustrated in Fig 5G).

### Depletion of MALAT1 enhances Smads-mediated iPS cell differentiation into hepatocyte-like cells

**Fig 6:**
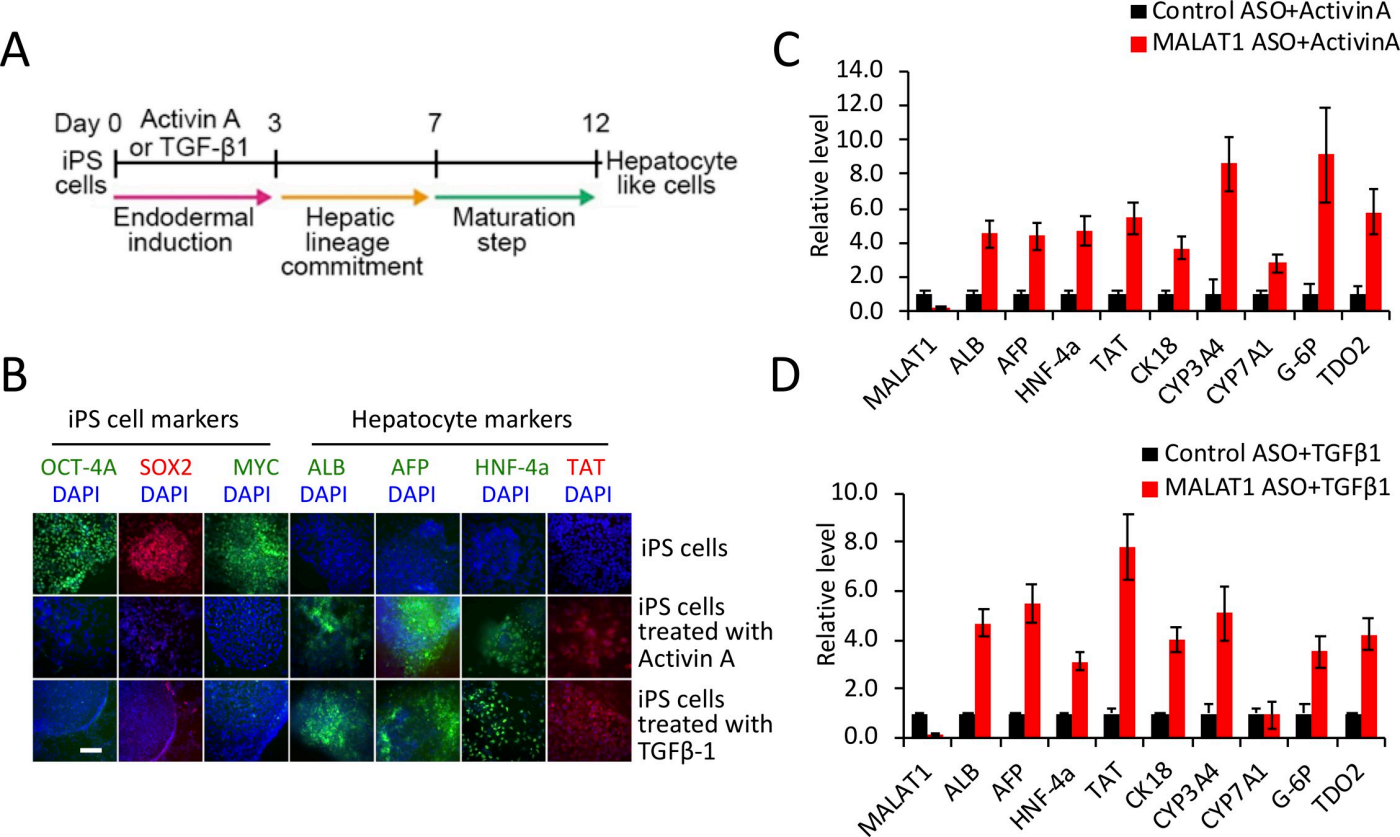
MALAT1 depletion enhances TGF-β- and Activin A-induced iPS cell differentiation to hepatocytes. **A,** A schematic representation of three-step induction from iPS cells to hepatocyte-like cells. **B,** Immunofluorescence staining for iPS cell markers (OCT-4A, SOX2, MYC) and hepatocyte markers (ALB, AFP, HNF-4a and TAT) before and after hepatic lineage induction (scale bar = 1mm). **C** and **D,** Quantitative RT-PCR analyses for the hepatocyte markers ALB, AFP, HNF-4a, TAT, CK-18, CYP3A4, CYP7A1, G-6P, and TDO2 in cells with or without MALAT1 depletion. iPS cells were transfected with MALAT1-specific 2’-OME-gamper ASO (phosphorothioated 2' O-methyl modified antisense oligonucleotide) or scramble control ASO; 24 hours later the cells were treated with Activin A **(C)** or TGF-β **(D)** for hepatocytes lineage induction as described in the Methods section. The data are shown as mean ± SD (n = 3).

Differentiation of induced pluripotent stem (iPS) cells into hepatocytes is an intriguing phenomenon in liver biology which strictly depends on Smads signaling[19, 30]. In this process, activation of Smad2/3 (by Activin A or TGF-β) is essential for endodermal induction and subsequent hepatocyte lineage commitment and maturation[31, 32]. In the context of Activin A, it belongs to the TGF-β superfamily and regulates gene transcription through activating Smad2/3 signaling pathway[33]. To explore the biological function of MALAT1-regulated Smad2/3 signaling, we performed further studies to determine whether MALAT1 might influence iPS cell differentiation into hepatocyte-like cells. The differentiation protocol used in this study was outlined in Fig 6A. After 12 days of differentiation, cells derived from iPS cells were analyzed for hepatocytes-specific markers by immunofluorescence staining. As shown in Fig 6B, the expression of iPS cell markers (OCT-4A, SOX2 and c-MYC) in hepatocyte-like cells were reduced, while the hepatocyte specific proteins ALB, AFP, HNF-4a and TAT were induced. These findings indicate successful induction of iPS cell differentiation into hepatocyte-like cells in our system. To assess the effect of MALAT1 on the hepatocyte differentiation process, we transfected the iPS cells with MALAT1-specific 2’-OME-gamper ASO1 (phosphorothioated 2' O-methyl modified antisense oligonucleotide) or scramble control 2’-OME-gamper ASO (24 hours prior to Activin A or TGF-β incubation); at the end of the induction/differentiation process, the expression of hepatocyte specific markers were assessed by qRT-PCR. Our data showed that depletion of MALAT1 enhanced the expression of hepatocyte-specific genes (including ALB, AFP, HNF-4a, TAT and others) in cells incubated with either Activin A (Fig 6C) or TGF-β (Fig 6D).

## Discussion

The canonical TGF-β/Smads signaling pathway in cells is finely regulated at different levels, including sequential activation of TGFβRII and TGFβRI through ligand binding, the phosphorylation of Smad proteins, regulation by inhibitory Smads, regulation of Smads activity in nucleus by kinases and phosphatases, proteasome for Smads degradation, and a group of microRNAs that target different components of the TGF-β pathway. In the current study, we describe a novel mechanism through which a long non-coding RNA regulates cellular TGF-β/Smads activity.

Our results presented in the current study show that MALAT1 is involved in the regulation of TGF-β/Smad pathway through formation of a lncRNA-protein complex with SETD2, pSmad2/3, and PPM1A. Specifically, the SETD2 protein mediates the association between MALAT1 and pSmad2/3; PPM1A is a nuclear Ser/Thr protein phosphatase, which dephosphorylates pSmad2/3 through physical interaction thus terminating Smad signaling [29]. Our data show that both SETD2 and PPM1A are associated with MALAT1 to assemble the scaffold for pSmad2/3 dephosphorylation. Our data support the concept that the PY motif (in pSmads) and the WW domain (in SETD2) are the physical basis for the binding of the two proteins; this interaction is essential for pSmad2/3 to be assembled into this lncRNA-protein dephosphorylation complex. Our findings are consistent with the previous report that linker region phosphorylation accelerates pSmad2/3 dephosphorylation and drives their turnover [22]. We show that PPM1A molecules are scattered throughout the nuclear envelope with only a portion overlapping with the nuclear speckle marker SC-35 and that TGF-β stimulation exhibits no effect on the level and localization of PPM1A. These findings are consistent with the notion that PPM1A is a phosphatase with multiple substrates in addition to pSmad2/3[34]. We noted that although MALAT1 depletion decreased SETD2 association with PPM1A and pSmad2/3, it had little effect on its other functions, such as histone H3K36 methyltransferase activity.

As a highly conserved nuclear noncoding RNA, MALAT1 was first described as tumor metastasis related gene in non-small cell lung cancer [17]. Recent studies showed that MALAT can bind to active chromatin sites[35] and regulate gene transcription[14, 15] and precursor mRNA splicing[16]. MALAT1 is localized in nuclear speckles, a dynamic structure containing pre-mRNA splicing factors and transcription factors[36]; this special localization provides the basis for physical interaction between MALAT1 and some transcription factors, such as pSmad2/3. Considering its broad expression spectrum and high conservation among multiple mammalian species, MALAT1 is believed to have fundamental cytological and genetic functions[37]. One study identifies 22 genes with altered expression in the livers of male MALAT1 knockout mice[15]. PAI-1, whose transcription is activated by Smad signaling, was found to be one of the most increased expressed genes. Through analyzing promoter regions (from -700 to +299) of those 22 genes, we found 13 of them contain 3 or more Smad Binding Element (SBE) core sequence (5’-CAGAC-3’ or 5’-GTCTG-3’)[9] in their promoter. Miyagawa et al[38] show that MALAT1 depletion or delocalization decreases the expression of 2′-5′-oligoadenylate synthetase like protein (OASL), interferon-induced protein 44 (IFI44), and serine peptidase inhibitor Kazal type 4 (SPINK4) in Hela cells; those genes or their family members are known to be involved in interferon and TGF-β associated lung fibrosis[39]. Another interesting study by Watts et al[13] shows that myostatin, a member of the TGF-β superfamily, decreases MALAT1 more than 10 folds through non-Smad pathway, which, in turn, enhances its myogenesis-inhibitory function via the canonical TGF-β signaling pathway in human skeletal muscle cells. All of these findings support an important link between MALAT1 and TGF-β signaling pathway.

In the current study, we examined functional effect of MALAT1 on Smad2/3 activation by using the iPS cell-hepatocyte differentiation model, which is a well-established system to assess the activity of Smad singling pertinent to liver biology[19, 30–32]. By employing this system, we demonstrate that MALAT1 depletion enhances TGF-β or Activin A-induced Smad2/3 activity and increases the expression of hepatocyte-specific genes.

Taken together, our experimental findings described in this study provide novel evidence that MALAT1 is a key regulator that determines TGF-β/Smad signaling. As the roles of TGF-β/Smads in cells are multifaceted and complex, further studies are warranted to assess the impact of MALAT1 on TGF-β/Smad signaling in other cell types and tissues.

## Supporting information

S1 File Supplementary materials and methods. (PDF)

S2 File Supplementary figures. (PDF)

S1 Raw Images (PDF)

## Abbreviations

LncRNAs: long noncoding RNAs
MALAT1: metastasis associated lung adenocarcinoma transcript 1
SETD2: SET domain containing 2
PPM1A: protein phosphatase, Mg2+/Mn2+ dependent, 1A
RIP: RNA immunoprecipitation
KLF4: Kruppel-like factor 4
OCT4A: octamer-binding transcription factor 4a
SOX2: SRY (sex determining region Y)-box 2
AFP: alpha-fetoprotein
ALB: albumin
CK18: cytokeratin 18
CYP3A4: cytochrome P450 3A4
CYP7A1: cytochrome P450 7A1
G-6P: glucose-6-phosphatase
HNF-4a: hepatocyte nuclear factor-4a
TAT: tyrosine aminotransferase
TDO2: tryptophan 2,3-dioxygenase
