# Supplementary material for: The long-noncoding RNA MALAT1 regulates TGF-β/Smad signaling through formation of a lncRNA-protein complex with Smads, SETD2 and PPM1A in hepatic cells
Source: PLoS One. 2020 Jan 29;15(1):e0228160. doi: 10.1371/journal.pone.0228160 (PMC6988980; doi:10.1371/journal.pone.0228160)
Supplement: S1 File — (PDF) [file pone.0228160.s001.pdf]

Supplementary Materials and Methods

Oligonucleotides and primers sequences

Oligo/primers	Sequence (5' to 3')
**Oligonucleotides**	
MALAT1-shRNA	TCCTTGGGACGCAGCGACGAGTTGTGCTG
MALAT1-AS1	mG*mG*mG*mA*mG*T*T*A*C*T*T*G*C*C*A*mA*mC*mU*mU*mG
MALAT1-AS2	mA*mU*mG*mG*mA*G*G*T*A*T*G*A*C*A*T*mA*mU*mA*mA*mU
Scrambled	mU*mC*mA*mC*mC*T*T*C*A*C*C*C*T*C*T*mC*mC*mA*mC*mU*
SBE-sense	TCGATAGCCAGACAGGTAGCCAGACAGG
SBE-antisense	CCTGTCTGGCTACCTGTCTGGCTATCGA
**Primers**	
MALAT1-forward	TGTAGTTCAGTGTTGGGGCA
MALAT1-reverse	GCTCAAATCCTGATCTGGTCC
MEG3-forward	GCCCACGAGAGGATCCCTCACC
MEG3-reverse	CGTGCTCCTAGTGCCCTCGTGA
HOTAIR-forward	CGTGGCAGAGGGCAAGACGG
HOTAIR-reverse	GTGGCGAGCTAGGACCCGGA
HULC-forward	AGAAACTCTGAAGTAAAGGCCGGA
HULC-reverse	TGCCAGGAAACTTCTTGCTTGATGC
HEIH-forward	CCTCTTGTGCCCCTTTCTT
HEIH-reverse	ATGGCTTCTCGCATCCTAT
AFP-forward	GCAGCCAAAGTGAAGAGG
AFP-reverse	TGTTGCTGCCTTTGTTTG
Albumin-forward	GGCACAATGAAGTGGGTAAC
Albumin-reverse	AGGCAATCAACACCAAGG
CK-18-forward	CTACATCAACAACCTTAGGC
CK-18-reverse	TCCACATCCTTCTTGATG
G-6P-forward	TTCCCTGTAACCTGTGAGAC
G-6P-reverse	ATTCAAGCACCGAAATCTG
HNF-4a-forward	CCAAGTACATCCCAGCTTTC
HNF-4a-reverse	TTGGCATCTGGGTCAAAG
TDO2-forward	GGAACTACCTGCATTTGG
TDO2-reverse	TCTCTGAAGTCATTGAAGTCC
TAT-forward	ACTGTGTTTGGAAACCTGCC
TAT-reverse	GCAGCCACTTGTCAGAATGA

CYP3A4-forward	CCTTACATATACACACCCTTTG
CYP3A4-reverse	GGTTGAAGAAGTCCTCCTAAGCT
CYP7A1-forward	CTGCCAATCCTCTTGAGTTCC
CYP7A1-reverse	ACTCGGTAGCAGAAAGAATACATC
FOXA2-forward	TGCACTCGGCTTCCAGTATG
FOXA2-reverse	CATGTTGCTCACGGAGGAGT
GATA4-forward	GAACCTCCAGGCCCAGC
GATA4-reverse	TCTCTCCCTCGCAGGTCAAG
GAPDH-forward	GAGTCCACTGGCGTCTTC
GAPDH -reverse	GACTGTGGTCATGAGTCCTTC

Note: m: 2'O-Methyl bases; *: phosphorothioate bonds

Transfections with plasmids and oligonucleotides

Plasmids pGFP-V-RS-shMALAT1, pCMV6-AC-PPM1A, and their respective control were obtained from OriGene (Rockville, MD); pReceiver-M06-SETD2 and control from GeneCopoiea (Rockville, MD); pCS2-Flag-Smad2, pCS2-Flag-Smad3, pCS2-Flag-Smad3EPSM, and pCMV5-Flag-Smad2EPSM from Addgene (Cambridge, MA). MALAT1 (ID: n272238) and SETD2 (ID: s26423) SilencerSelect siRNA were purchased from Ambion. Transfections were carried out by using Lipofectamine2000 (for plasmids) and Oligofectamine (for siRNA) (Invitrogen Carlsbad, CA) as per manufacture instructions. Phosphorothioated 2' O-methyl modified oligonucleotides were synthesized by IDT DNA (Coralville, IA) and transfected to iPS cells by using Lipofectamine2000 as described previously(1).

Cells proliferation WST1 assay

Cells were cultured in serum-free medium for 24 hours to synchronize cell cycle. 2×10^3 cells were seeded into each well of 96-well plates in 200µl culture medium containing 1% fetal bovine serum. The medium was removed at indicated time points; 90µl serum-free medium mixed with 10µl WST-1 (Roche) was added to each well. Four hours after incubation, absorbance of each well was measured by an ELISA plate reader at wavelength of 450nm.

Luciferase activity assay

Twenty-four hours after p3TP-lux plasmid (Addgene) transfection, cells were incubated with TGF-β1 (5ng/mL) for additional 24 hours. The cells were then collected and analyzed by using the Dual-Luciferase reporter assay system (Promega, Madison, WI) as per user manual. Luciferase activity was measured by centro XS3LB960 microplate fluorescence reader (Berthold Tech, Ontario Canada). The pRL-TK plasmid with constitutive expression of Renilla luciferase was co-transfected with p3TP-lux as internal control.

Quantitative real-time PCR

Total RNA was prepared using Trizol reagent (Invitrogen). Reverse-transcription and quantitative PCR were performed by using SuperScript II Reverse Transcriptase (Invitrogen) and SYBR Green Supermix reagent (Qiagen, Valencia, CA).

Immunoprecipitation (IP)

The cells cultured in 100-mm dishes were fixed by 1% paraformaldehyde and then lysed in 1 ml RIPA buffer (1% Triton X-100, 0.1% sodium dodecyl sulfate, 50mM Tris-HCl pH 7.4, 150mM sodium chloride, 0.5% sodium deoxycholate, 1mM EDTA) containing phosphatase and protease cocktail inhibitors. Pre-cleared supernatants were rotated with 1μg antibodies and 50μl protein A/G-plus agarose beads at 4°C overnight; then samples were collected by centrifugation at 4°C 5000 rpm for 5 min, followed by five times wash with RIPA buffer; pellets were suspended in 2× SDS-PAGE sample loading buffer and subjected to Western blotting.

DNA pull-down assay

The cells in 100-mm dishes were lysed in RIPA buffer containing phosphatase and protease inhibitors. The samples were precleared with Streptavidin-agarose resin (Thermo) for 1 hour. The precleared supernatant was then incubated with 1μg biotinylated double-stranded SBE (Smad Binding Element) and 10μg poly (dI-dC) at 4°C for 24 hours. DNA-protein complex was collected by streptavidin-agarose resin incubation at 4°C for 1 hour. The complex was washed, boiled and subjected to West blotting.

Western blotting

The logarithmically growing cells were lysed in RIPA buffer with phosphatase and protease inhibitors. Centrifuged cell lysates were denatured at 95°C 5 min and subjected to SDS-PAGE gel. The separated proteins were transferred onto nitrocellulose membranes (BioRad, Hercules, CA); the membranes were blocked in 5% non-fat milk for 1 hour, followed by primary incubation with primary antibodies at 4°C overnight. IRDye 800CW or 680LT labeled antibody (1:10000) were used as secondary antibodies. The membranes were scanned and quantified with The Odyssey® Infrared Imaging System (LI-COR Biosciences, Lincoln, Ne).

Fluorescent in situ hybridization (FISH)

Probe set containing 48 different oligonucleotides for MALAT1 was designed and synthesized by Biosearch Technologies (Novato, CA) based on principles described by Raj et al(2). Cells on cover slide were fixed with 1% formaldehyde for 10 minutes at room temperature and permeabilized with 70% ethanol at 4°C overnight, followed by washing with Wash buffer (10% formamide, 2× saline-sodium citrate). Then, the cells were incubated with FISH probe and primary antibody diluted in Hybridization buffer (10% Dextran sulfate, 10% deionized formamide) in a dark humidified chamber at 37°C overnight. After washing with Wash buffer twice, the cells were incubated with secondary antibody in hybridization buffer for 1 hour at 37°C, followed by triple washings with Wash buffer, DAPI (5 ng/mL) counterstaining, and mounting with anti-fluorescence medium.

Immunofluorescence (IF)

Cells growing on coverslips (70% confluence) were washed twice with cold phosphate buffered saline and fixed in 2% paraformaldehyde for 10 min, followed by penetrating with 0.1% Triton X-100 and blocking with 5% BSA. Subsequently, the cells were incubated with primary antibodies at 4°C overnight, followed by three washings and incubation with Alexa488 or Alexa594 labeled secondary antibodies. After counterstaining with DAPI (0.1μg/ml) for 1min, the coverslips were applied with mounting medium for fluorescence; images were captured using fluorescence microscope.

1. Ma Y*, et al.* (2010) High-efficiency siRNA-based gene knockdown in human embryonic stem cells. *Rna* 16(12):2564-2569.
2. Raj A, van den Bogaard P, Rifkin SA, van Oudenaarden A, & Tyagi S (2008) Imaging individual mRNA molecules using multiple singly labeled probes. *Nat Methods* 5(10):877-879.
