# Supplementary material for: The long-noncoding RNA MALAT1 regulates TGF-β/Smad signaling through formation of a lncRNA-protein complex with Smads, SETD2 and PPM1A in hepatic cells
Source: PLoS One. 2020 Jan 29;15(1):e0228160. doi: 10.1371/journal.pone.0228160 (PMC6988980; doi:10.1371/journal.pone.0228160)
Supplement: S2 File — (PDF) [file pone.0228160.s002.pdf]

Supplementary figures

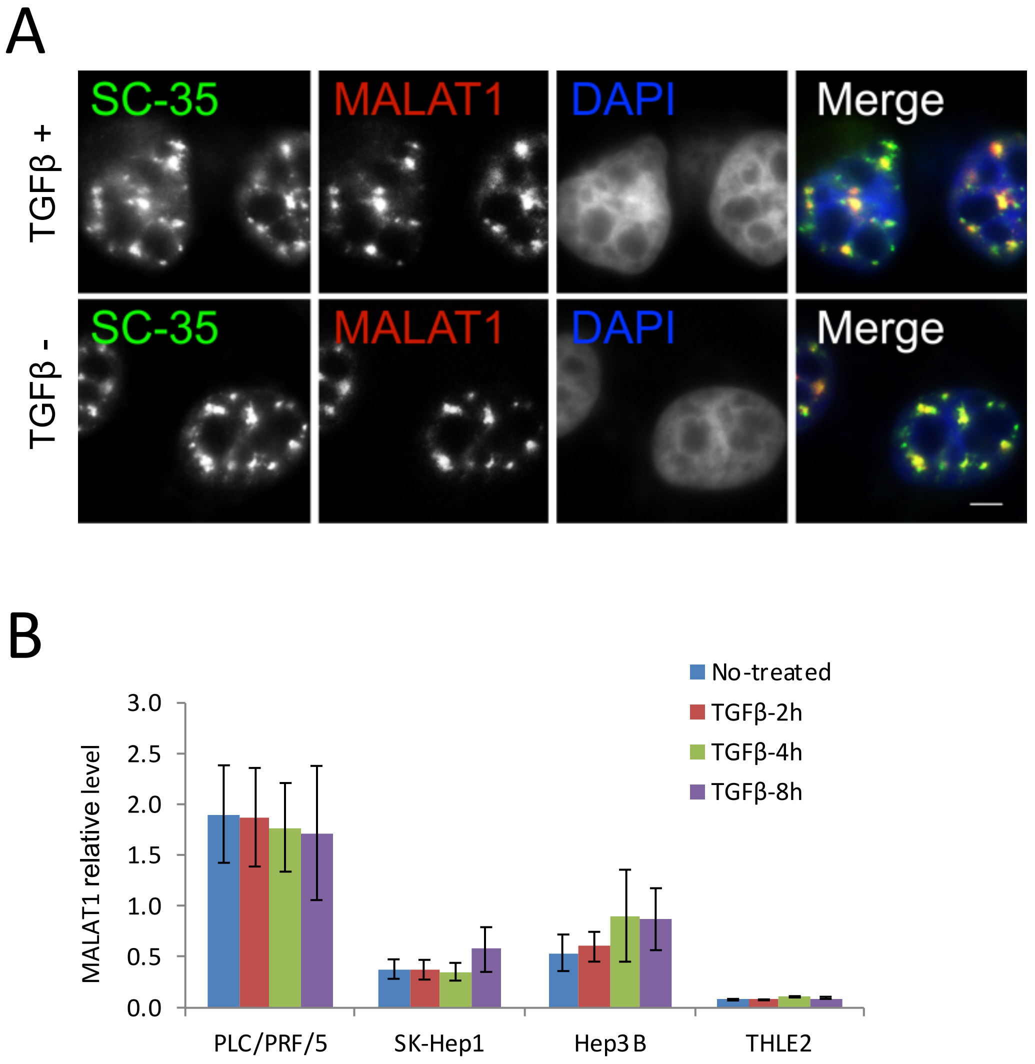

Figure S1. A. FISH-IF staining shows co-localization of MALAT1 and SC-35 (a nuclear speckle marker) in Hep3B cells with or without TGF-β1 treatment (5ng/ml of TGF-β1 for 1 hour), scale bar, 10µm. **B.** MALAT1 levels as determined by qRT-PCR in cells treated with TGF-β1 (5ng/ml) for 2, 4 or 8 hours. TGF-β1 treatment had no effect on MALAT1 levels in PLC/PRF/5, SK-Hep1, Hep3B and THLE2 cells. The qRT-PCR data were analyzed by ANOVA and the results are expressed as means ± s.d (n=5).

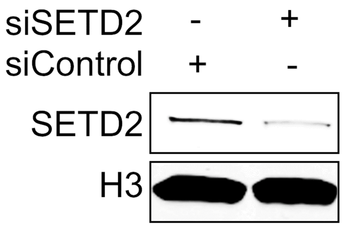

Figure S2. The efficacy of SETD2 knockdown by siRNA in Hep3B cells as determined by Western blot analysis.

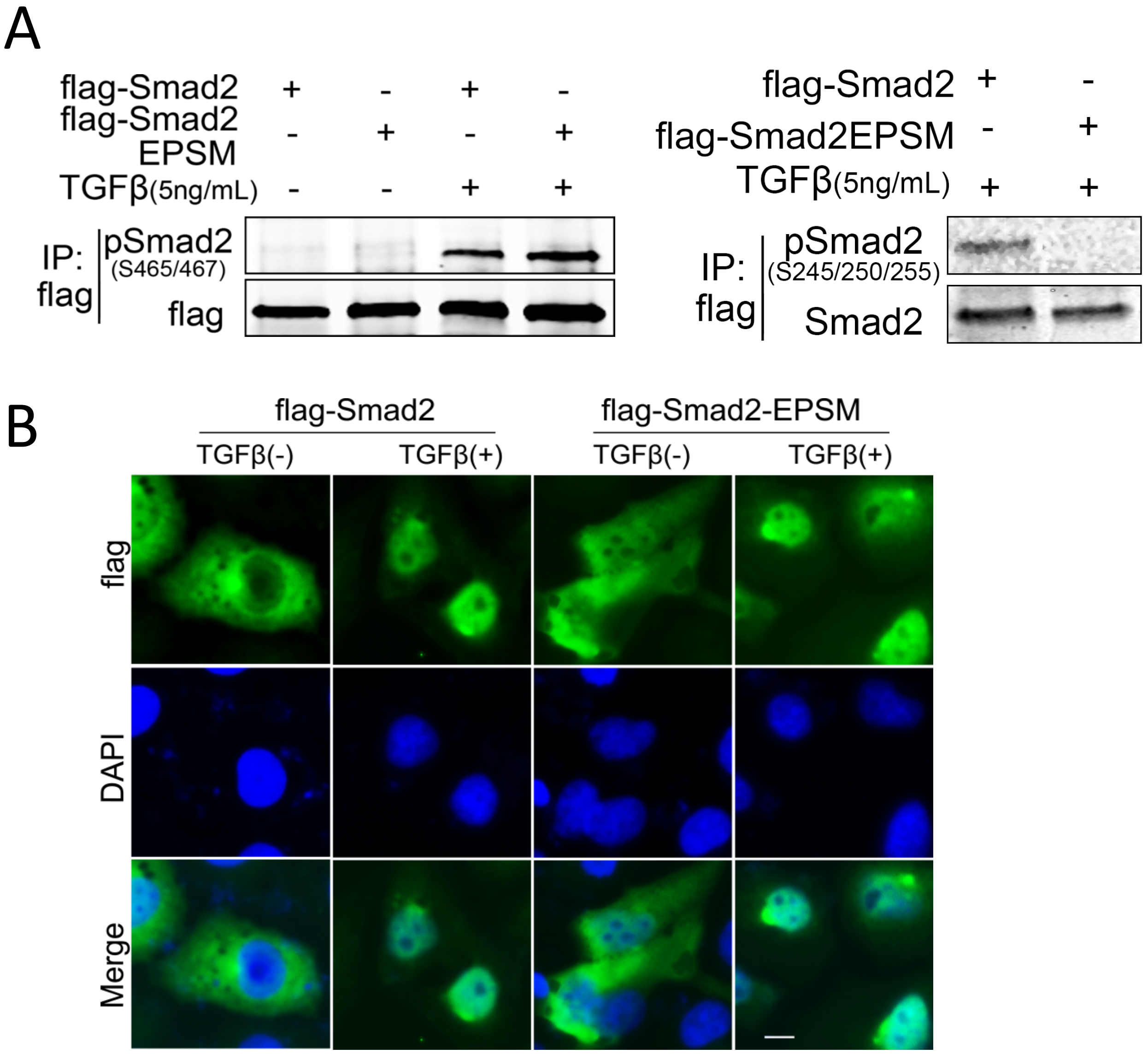

Figure S3. A. PLC/PRF/5 cells were treated with 5ng/ml TGF-β1 for 2 hours. Immunoprecipitation and Western blotting analyses were performed by using indicated antibodies. The C-terminal S465/467 phosphorylation in Smad2 and linker region mutated Smad2 (Smad2-EPSM) is shown in the left panel. The linker region S245/250/255 phosphorylation in Smad2 is shown in the right panel. **B.** IF shows translocation of Smad2 and Smad2-EPSM to the nuclei in PLC/PRF/5 cells (treated with 5ng/ml TGF-β1 for 1 hours), scale bar = 10 μm.

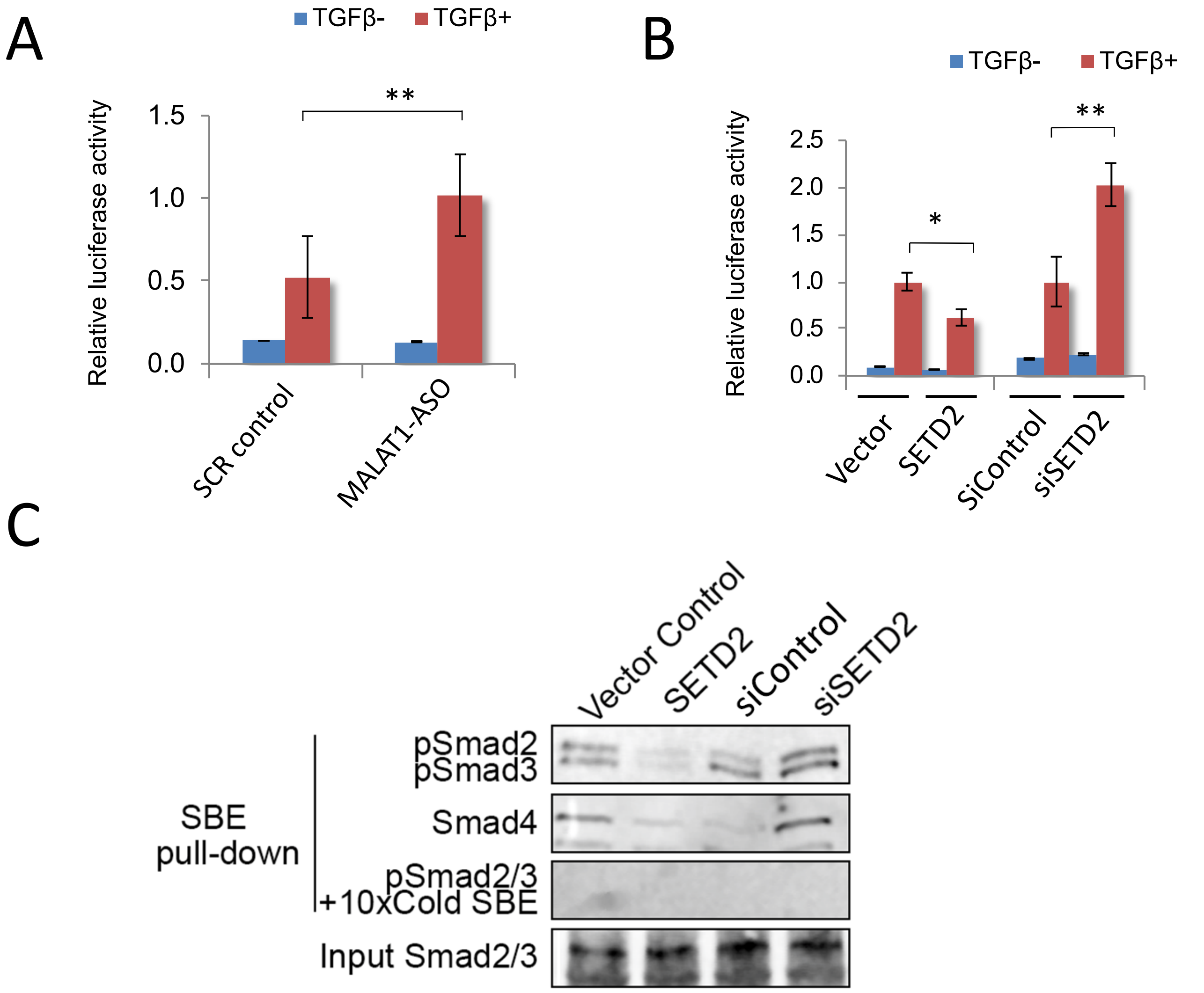

Figure S4. A. Hep3B cells were co-transfected with p3TP-lux reporter plasmid and MALAT1 specific or control 2'-MOE gapmer ASOs. Twenty-four hours after transfection, cells were stimulated with 5ng/ml TGF-β1 or vehicle for additional 24 hours. Then, the cells were lysed and subjected to dual-luciferase assay (n=3). Statistical analyses were performed by using a two tailed Student's t test method and the results are shown as mean ± s.d., $^{**}P < 0.01$. **B.** Hep3B cells were co-transfected with p3TP-lux reporter plasmid and SETD2 expression plasmid, siSETD2 or their corresponding controls. Twenty-four hours after transfection, cells were stimulated with 5ng/ml TGF-β1 or vehicle for additional 24 hours. The cells were then lysed and subjected to dual-luciferase assay (n=3). Statistical analyses were performed by using a two tailed Student's t test method and the data are shown as mean ± s.d., $^{*}P < 0.05$; $^{**}P < 0.01$. **C.** Cell lysates from Hep3B cells transfected with SETD2 overexpressed plasmids, siSETD2, or respective controls were subjected to DNA pull-down with biotinylated SBE probes followed by immunoblotting with indicated antibodies. The cells were stimulated with 5ng/ml TGF-β1 for 2 hours before being lysed.

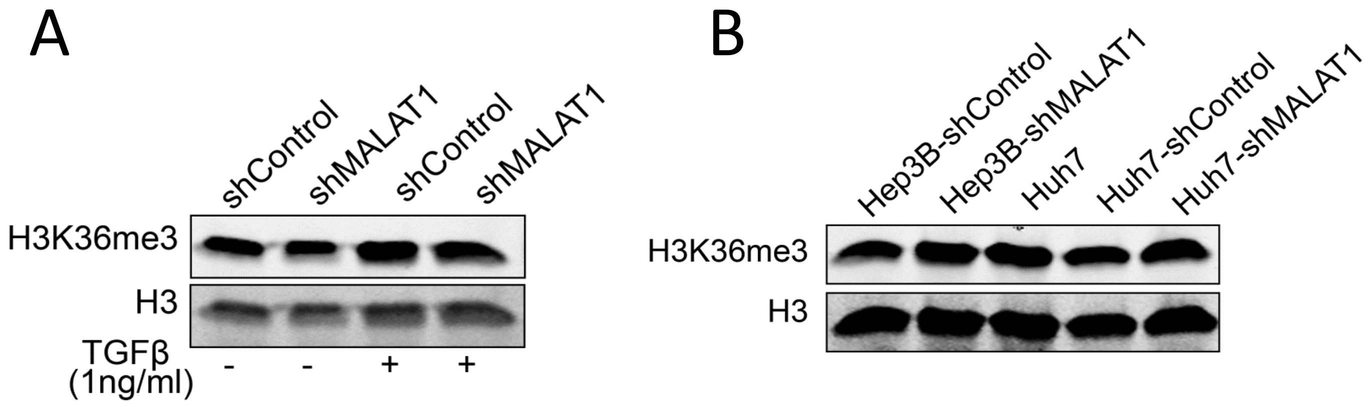

Figure S5. A. Immunoblotting for H3K36me3 in MALAT1 depleted or control Hep3B cells with or without TGF-β1 treatment (1ng/ml for 24 hours). **B.** Immunoblotting for H3K36me3 in Hep3B and Huh7 cells with or without MALAT1 depletion.

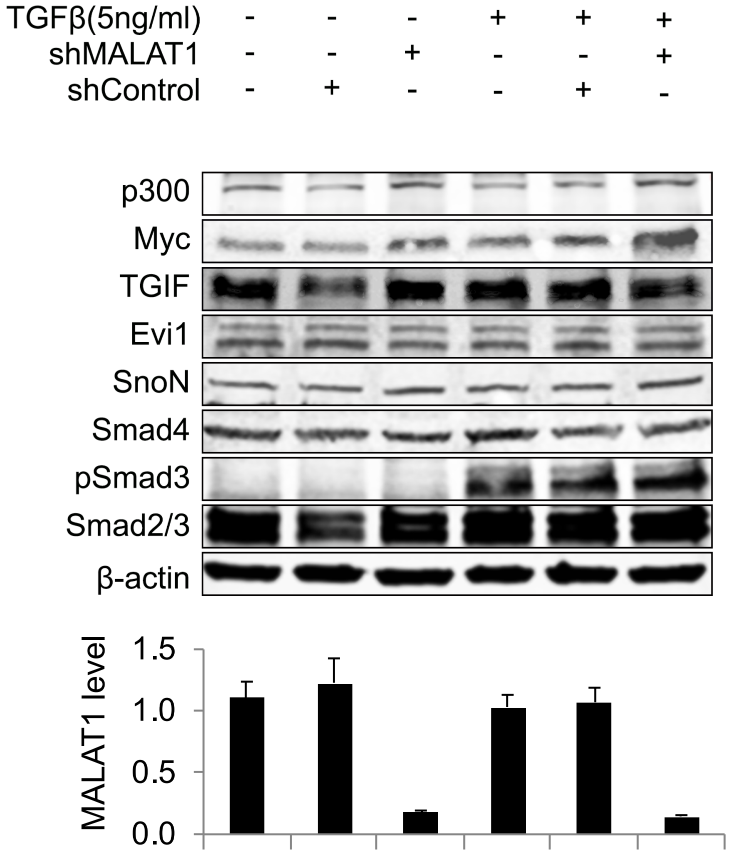

Figure S6. Immunoblotting results show the levels of indicated proteins in MALAT1 depleted and control Hep3B cells with or without TGF-β1 treatment (5ng/ml for 24 hours) (upper panel). The levels of MALAT1 in these cells were quantified by qRT-PCR (lower panel).

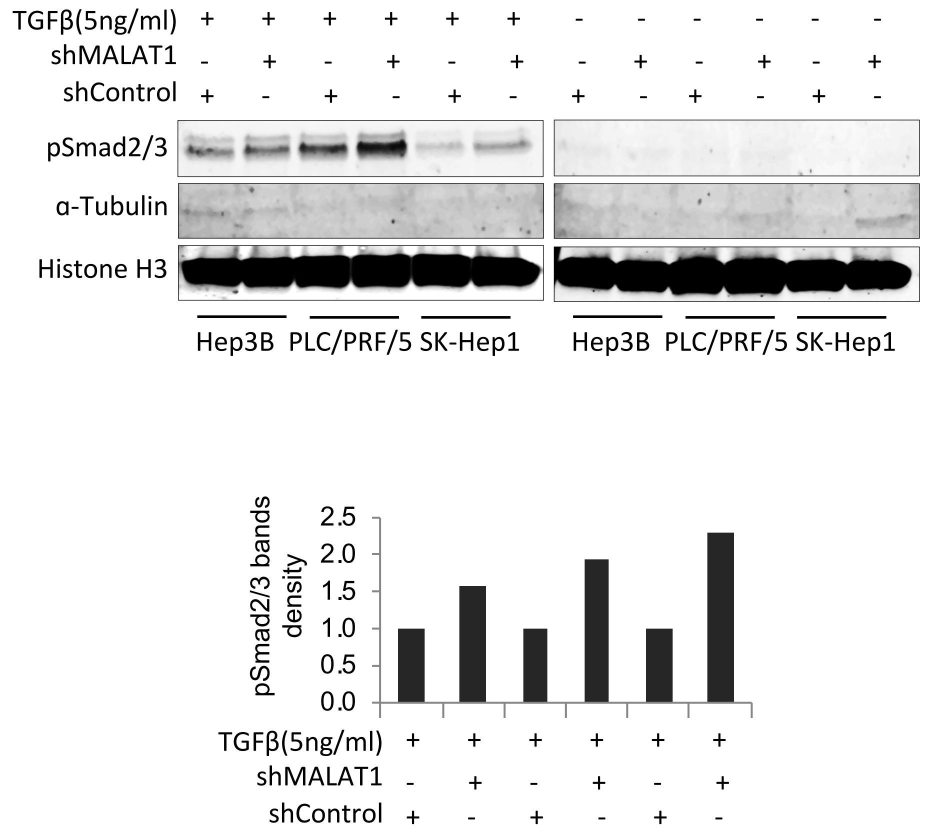

Figure S7. Immunoblotting for pSmad2/3 in Hep3B PLC/PRF/5 and SK-Hep1 cells with or without TGF-β1 treatment (for 2 hours). Nuclear proteins were obtained for Western blotting analysis using antibodies against pSmad2/3. Immunoblotting for α-Tubulin was performed. The pSmad2/3 band densities from TGF-β1 treated cells were quantified by using Odyssey image software (Li-Cor Bioscience, Lincoln, NE).
