# Supplementary material for: The long-noncoding RNA MALAT1 regulates TGF-β/Smad signaling through formation of a lncRNA-protein complex with Smads, SETD2 and PPM1A in hepatic cells
Source: PLoS One. 2020 Jan 29;15(1):e0228160. doi: 10.1371/journal.pone.0228160 (PMC6988980; doi:10.1371/journal.pone.0228160)

Original images for Figure 2A

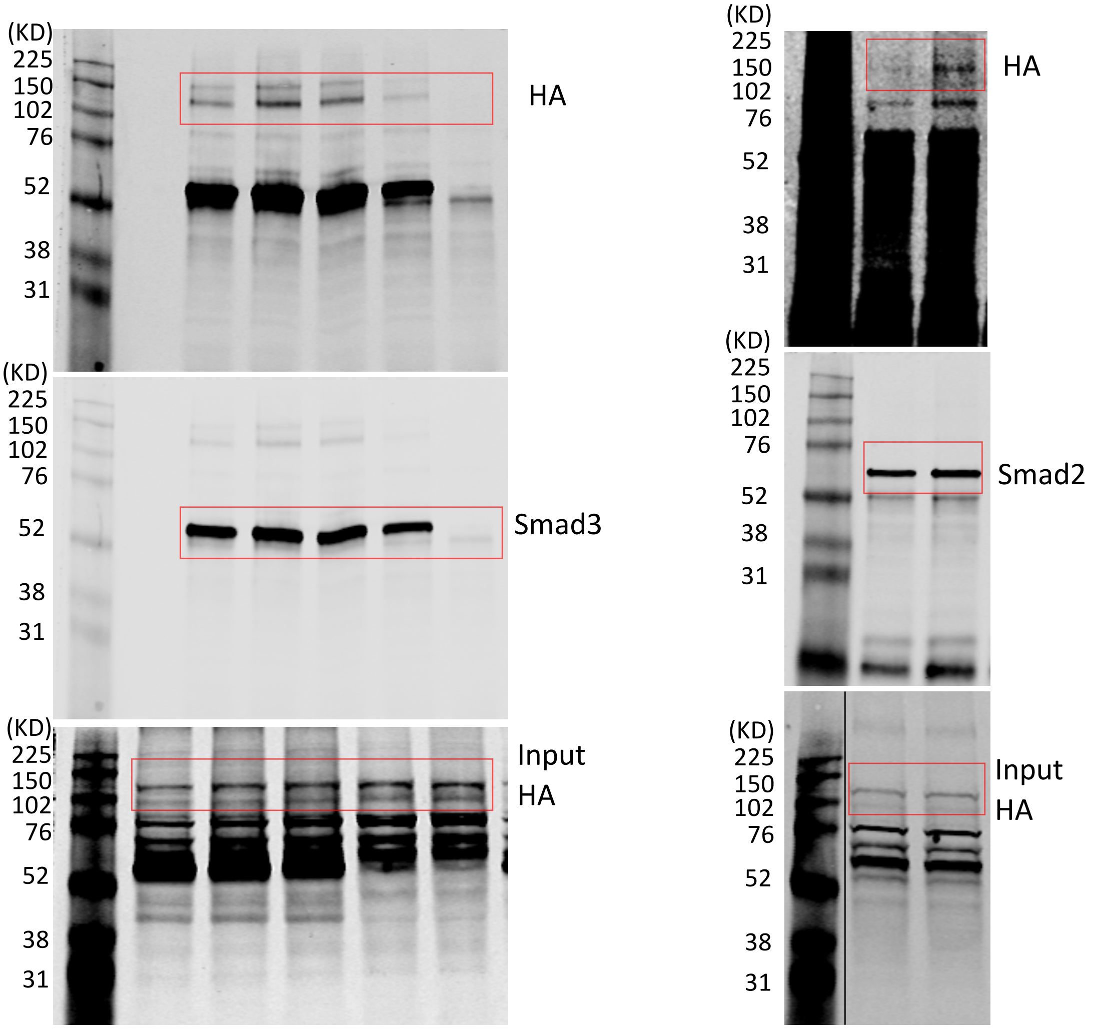

Original images for Figure 2D

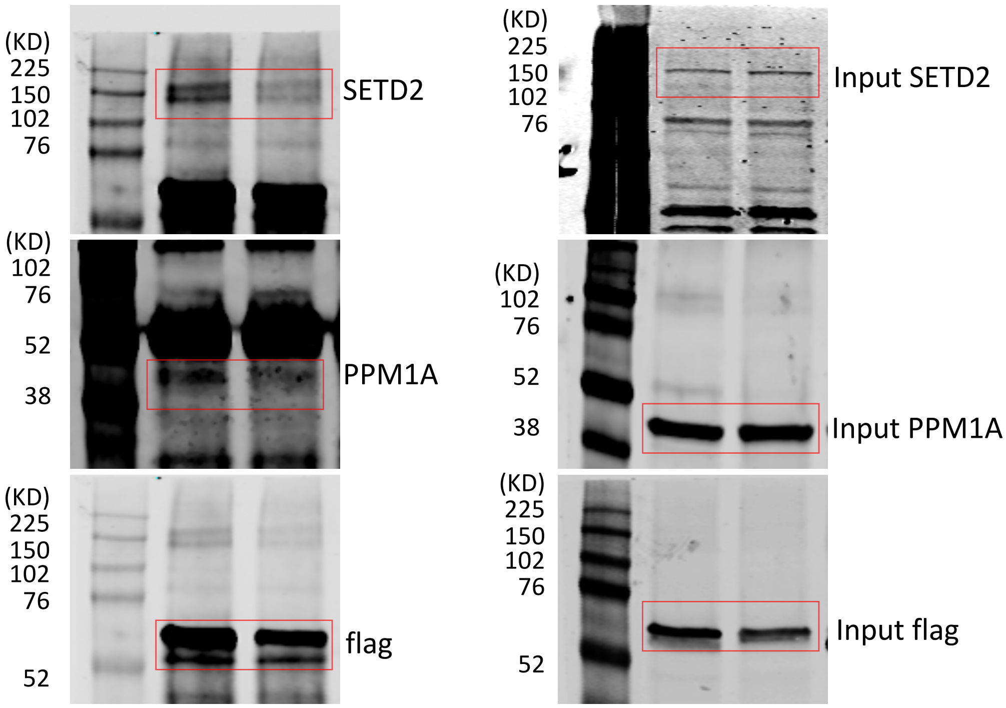

Original images for Figure 3C

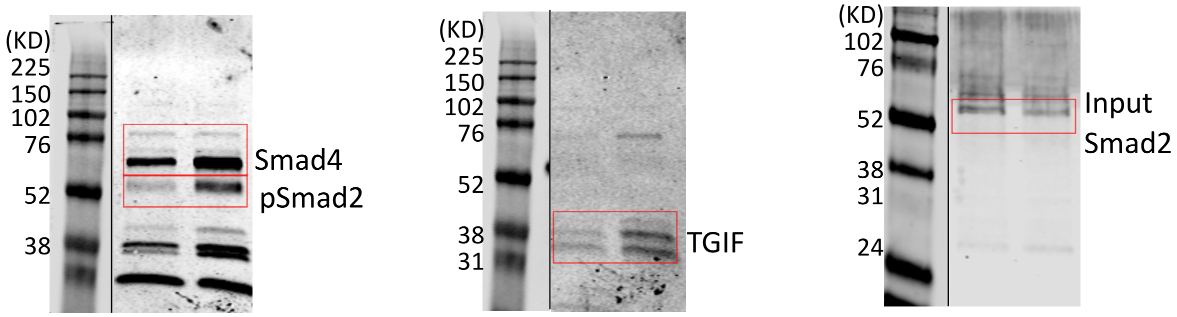

Original images for Figure 3D

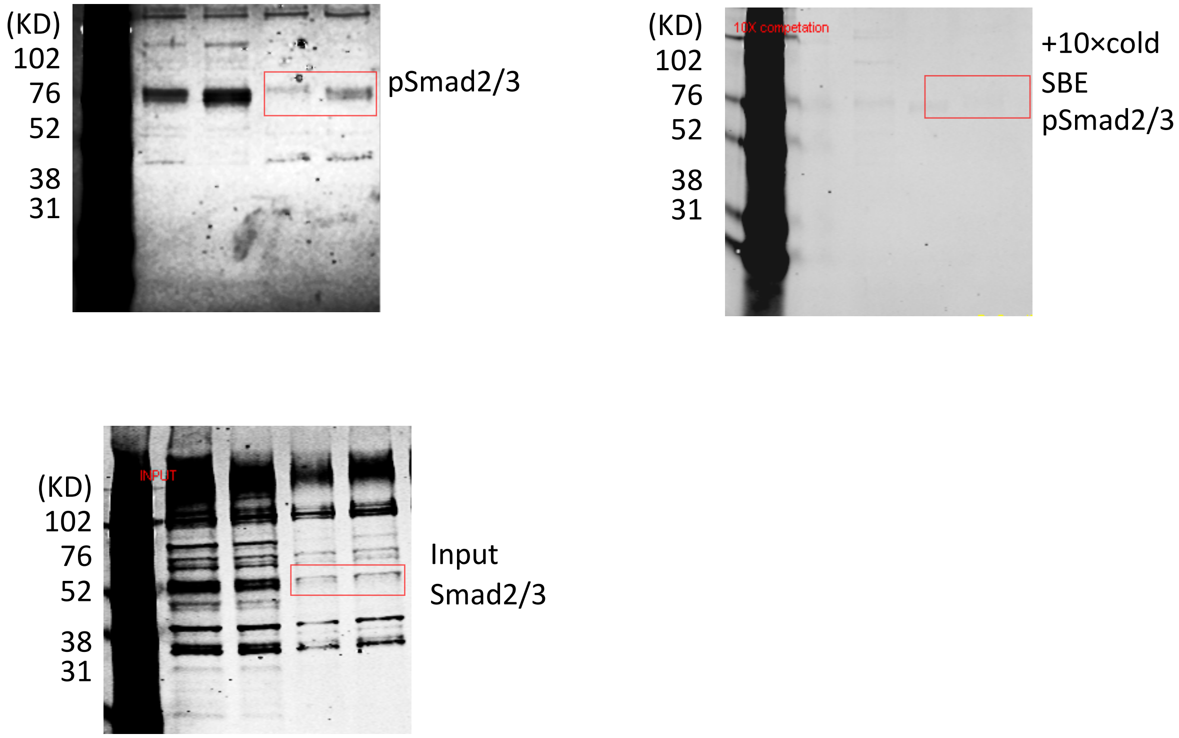

Original images for Figure 3E

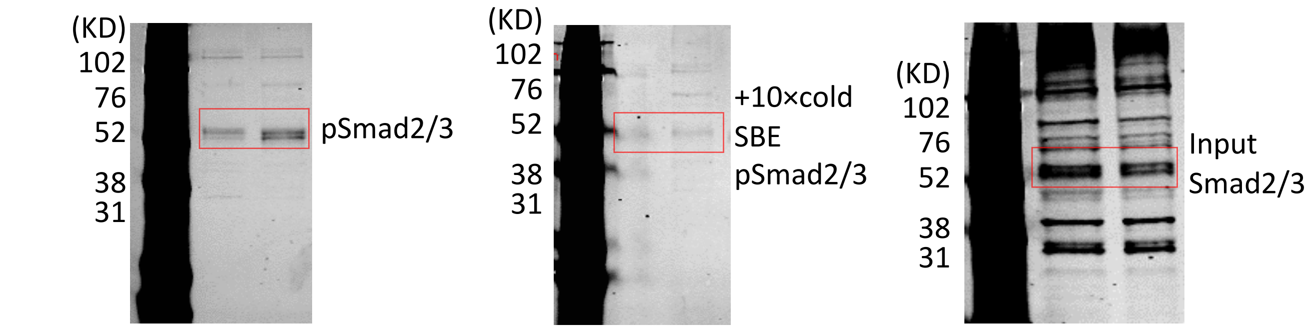

Original images for Figure 4A

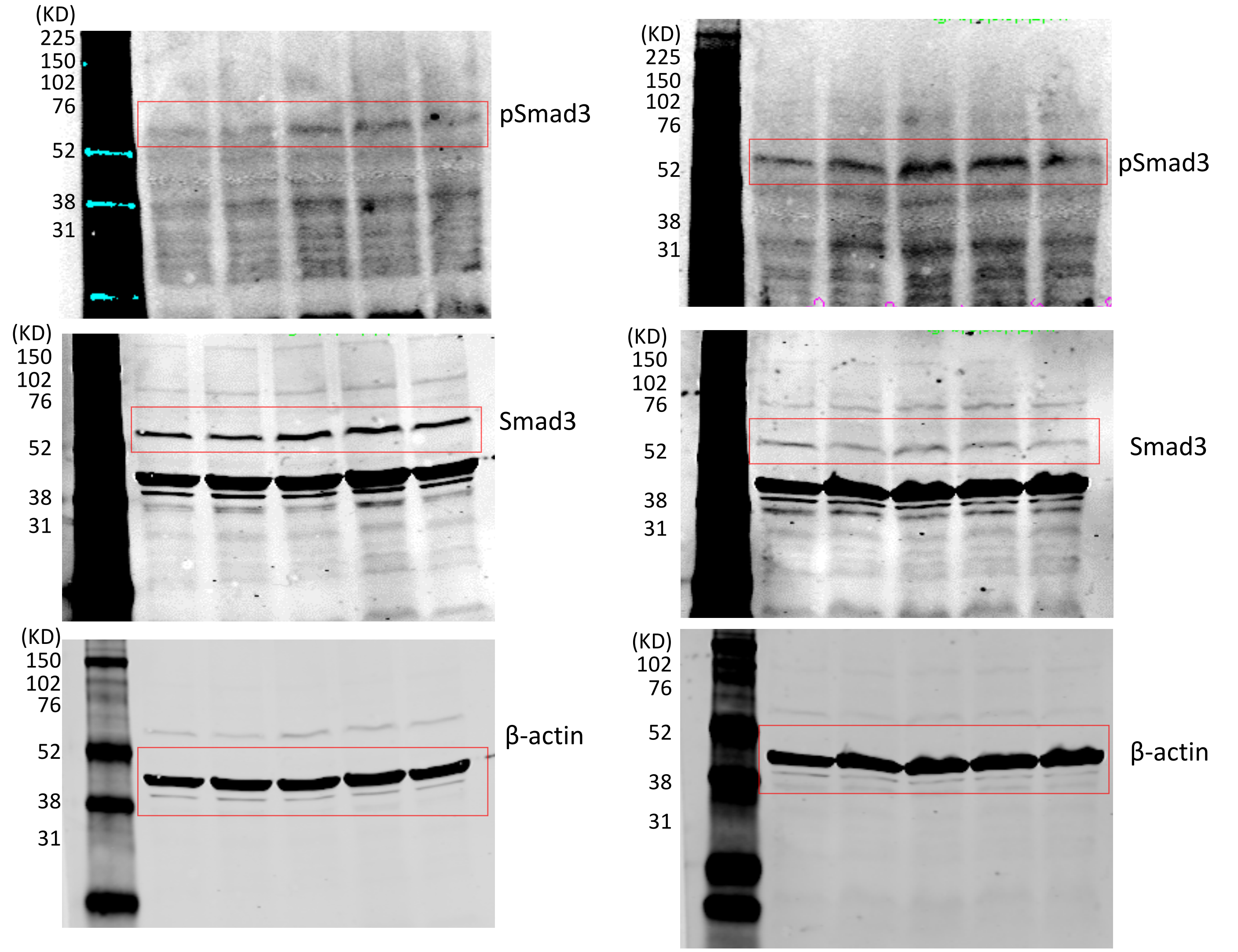

Original images for Figure 4C

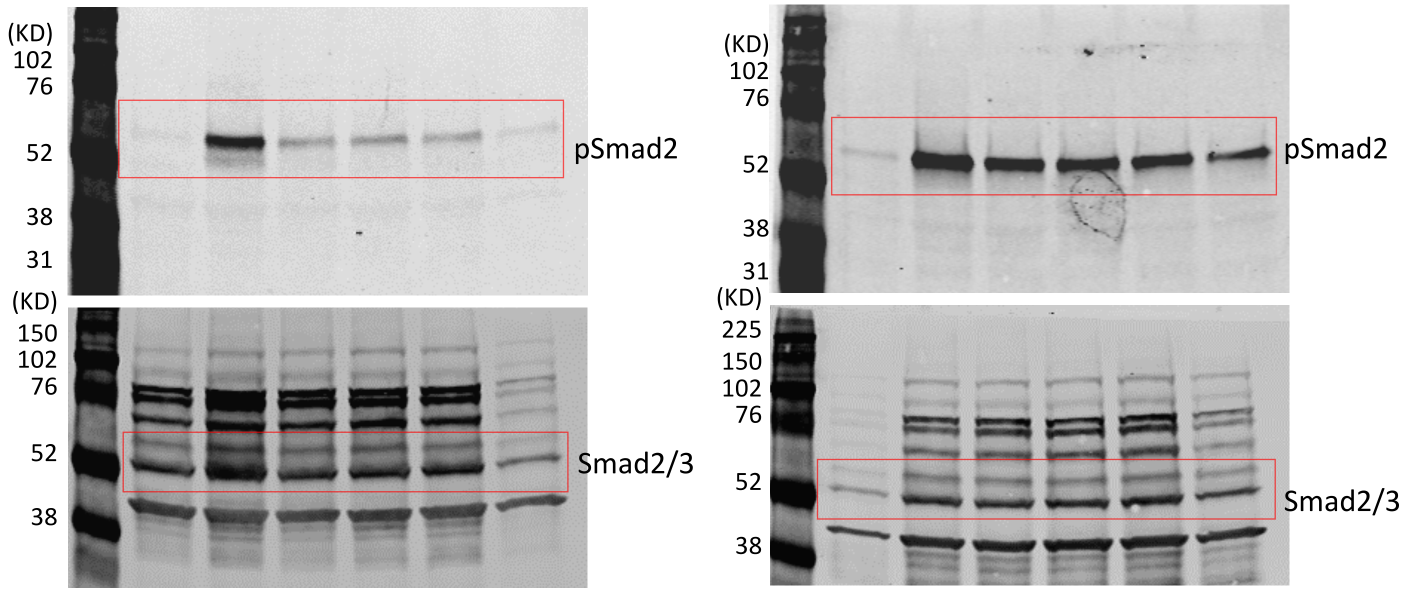

Original images for Figure 4E

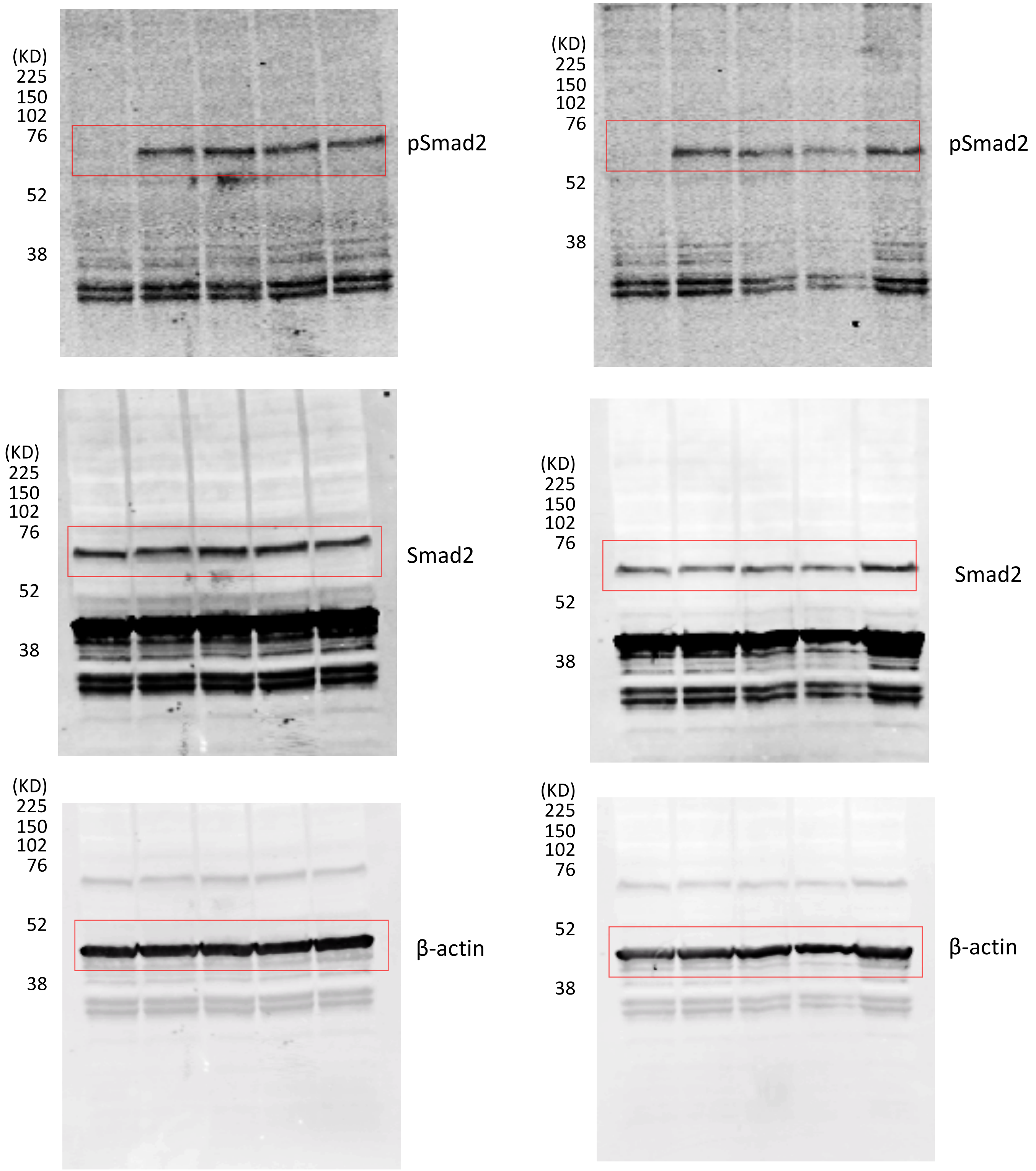

Original images for Figure 4G

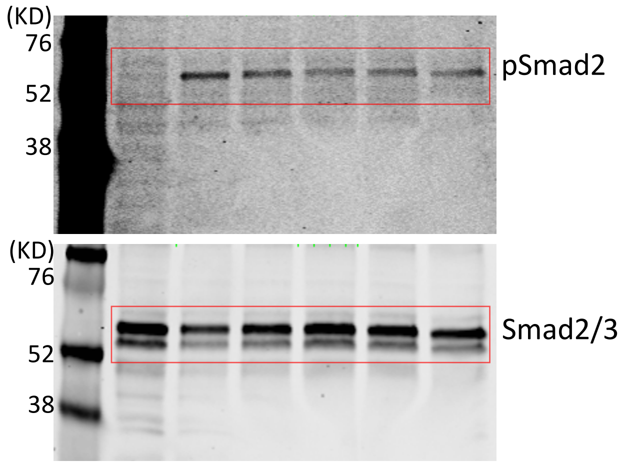

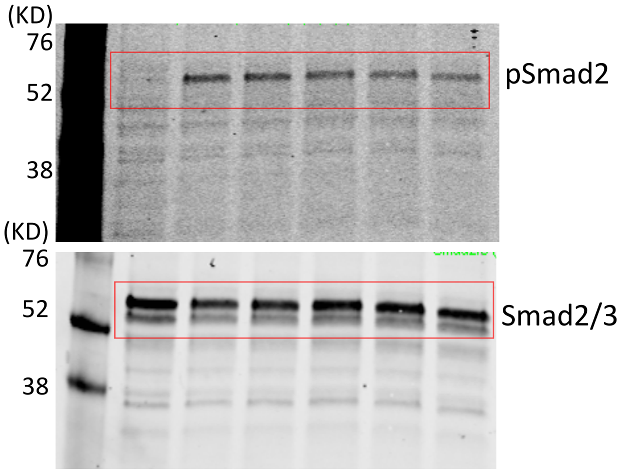

Original images for Figure 5A

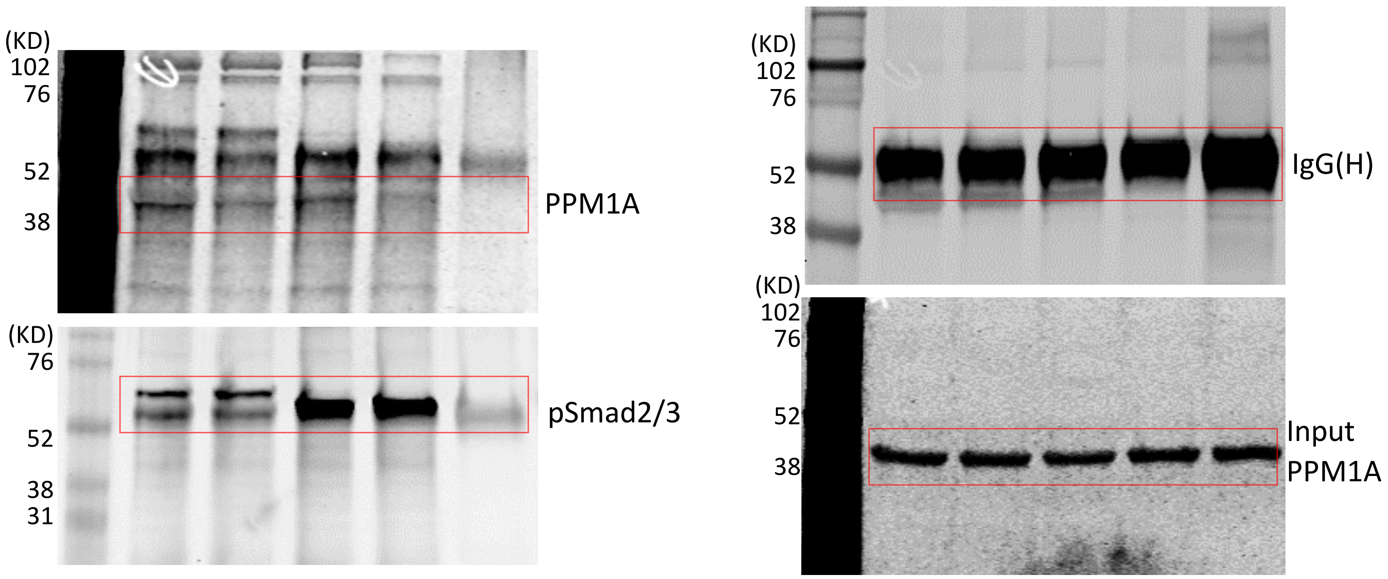

Original images for Figure 5B

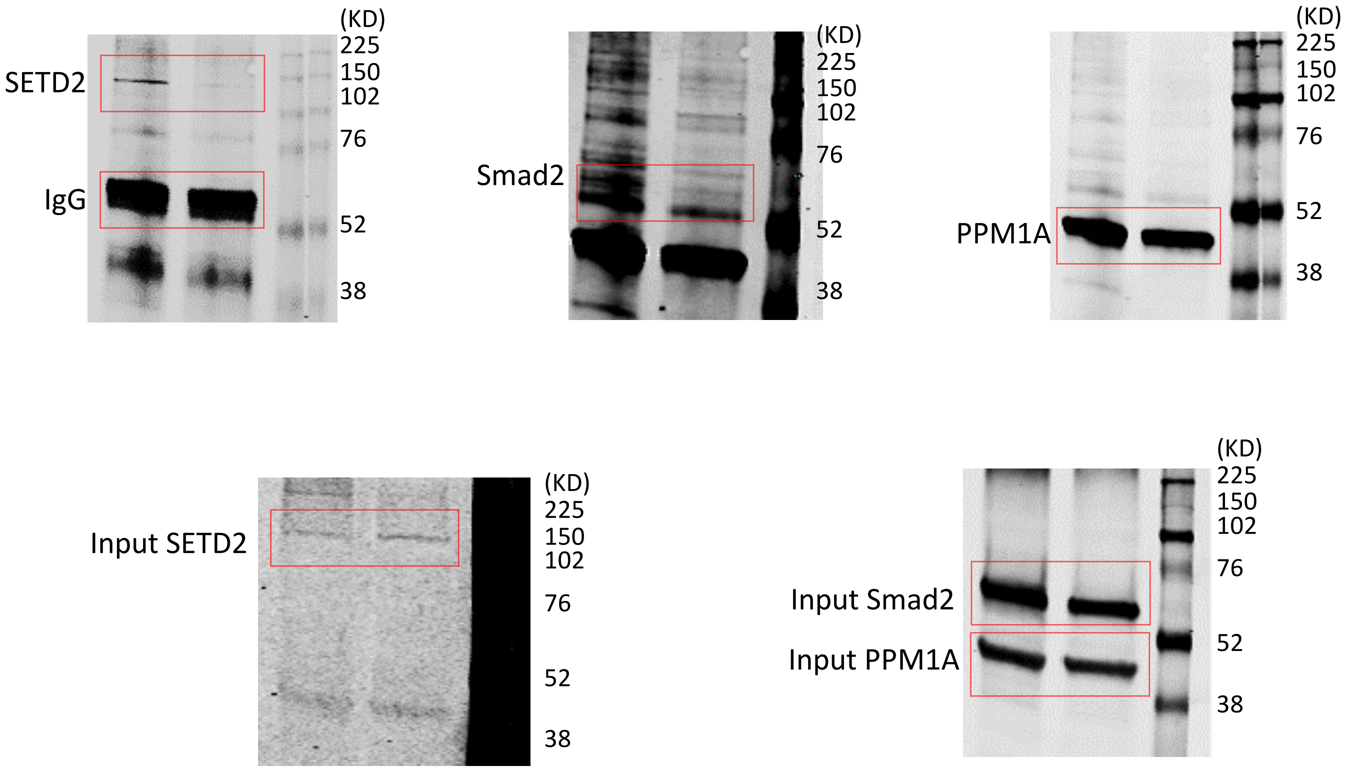

Original images for Figure 5C

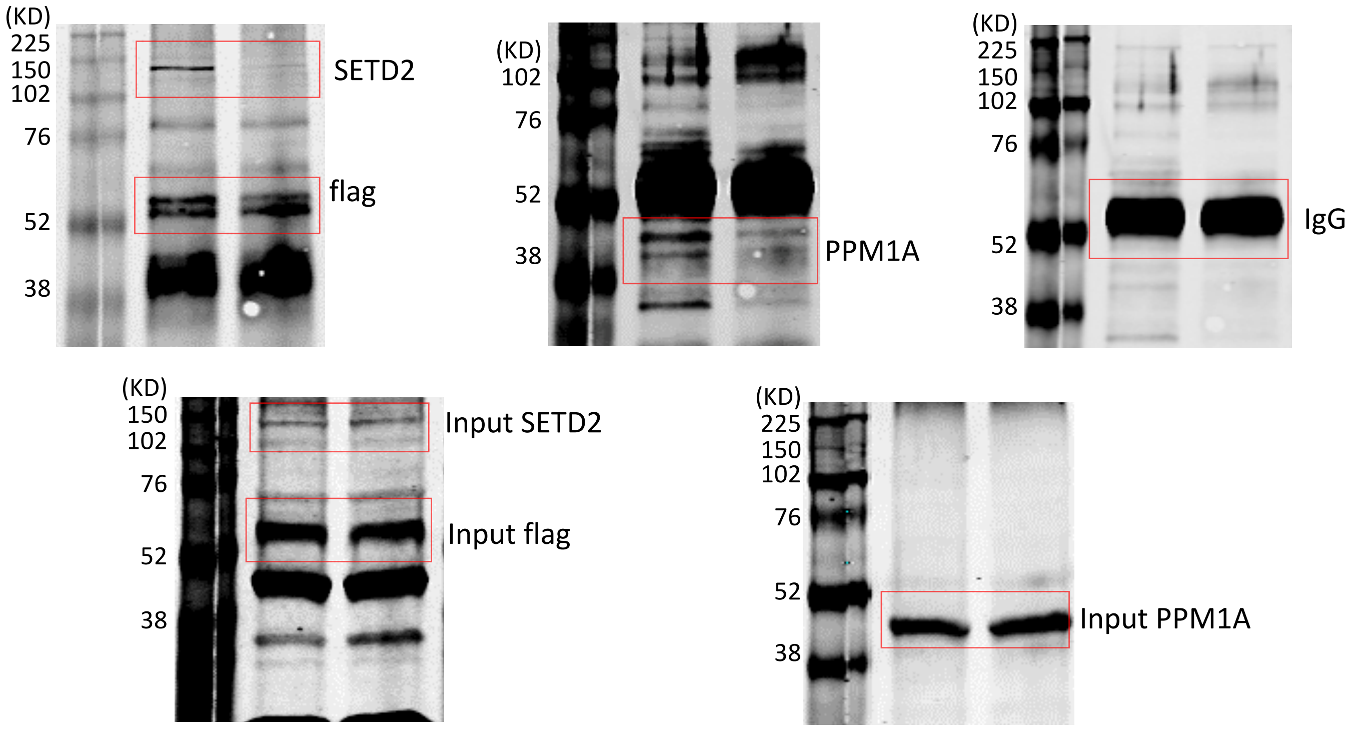

Original images for Figure 5D

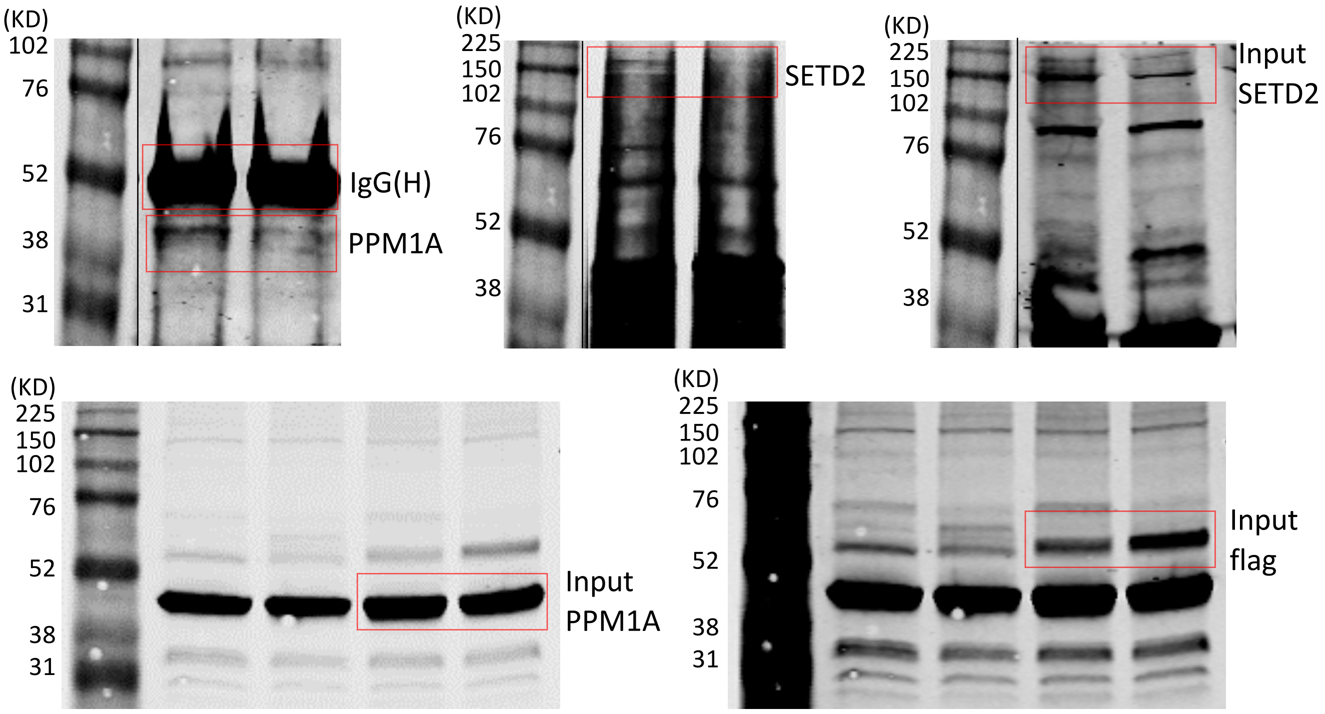

Original images for Figure 5F

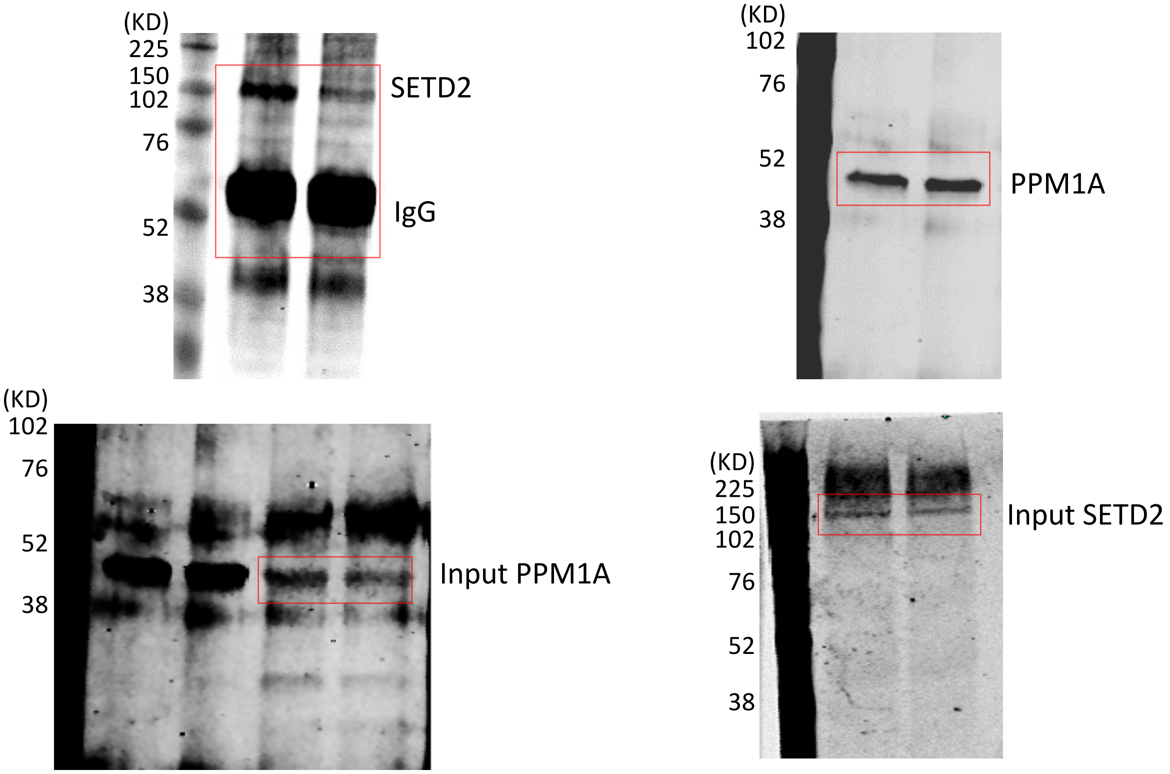

Original images for Figure S2

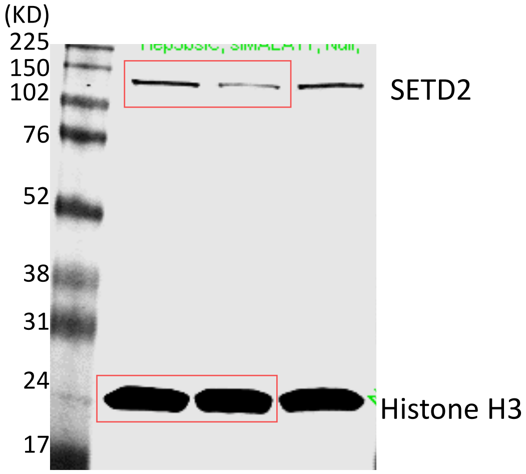

Original images for Figure S3A

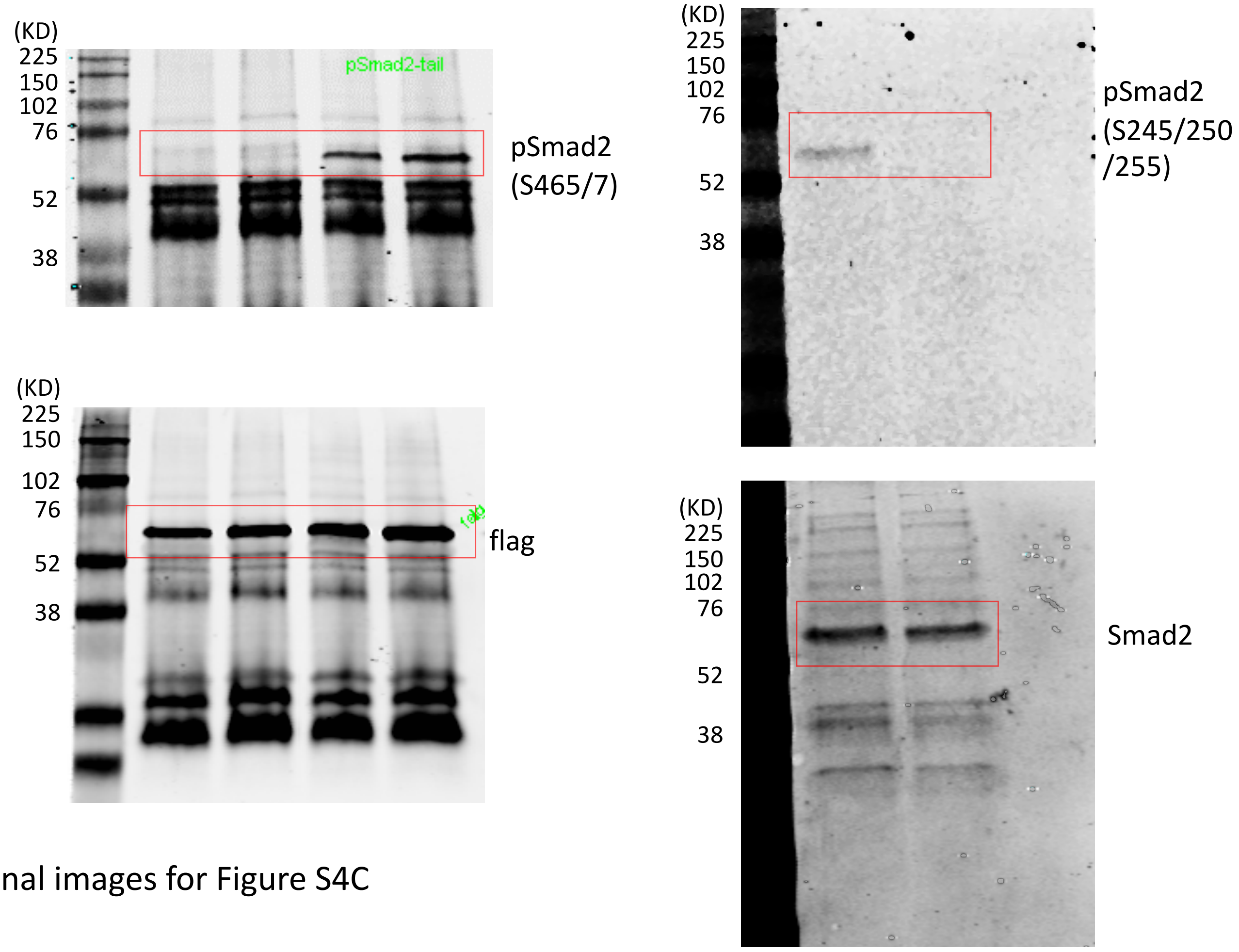

Original images for Figure S4C

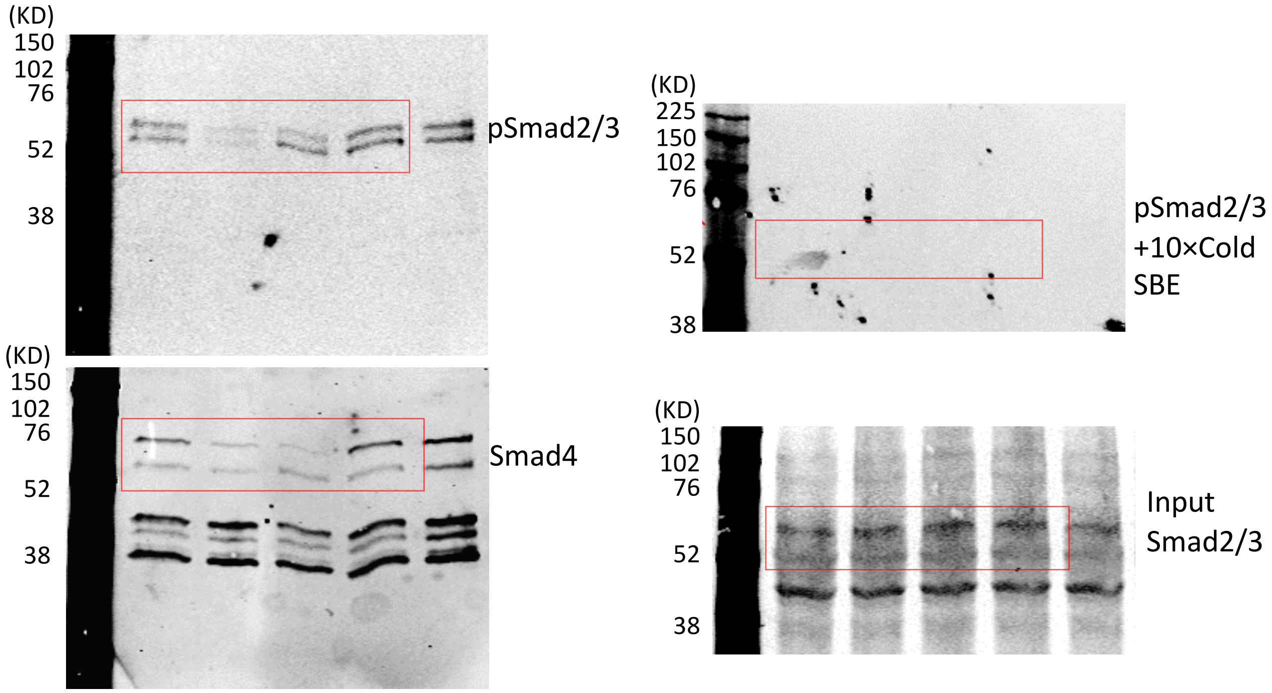

Original images for Figure S5A, S5B

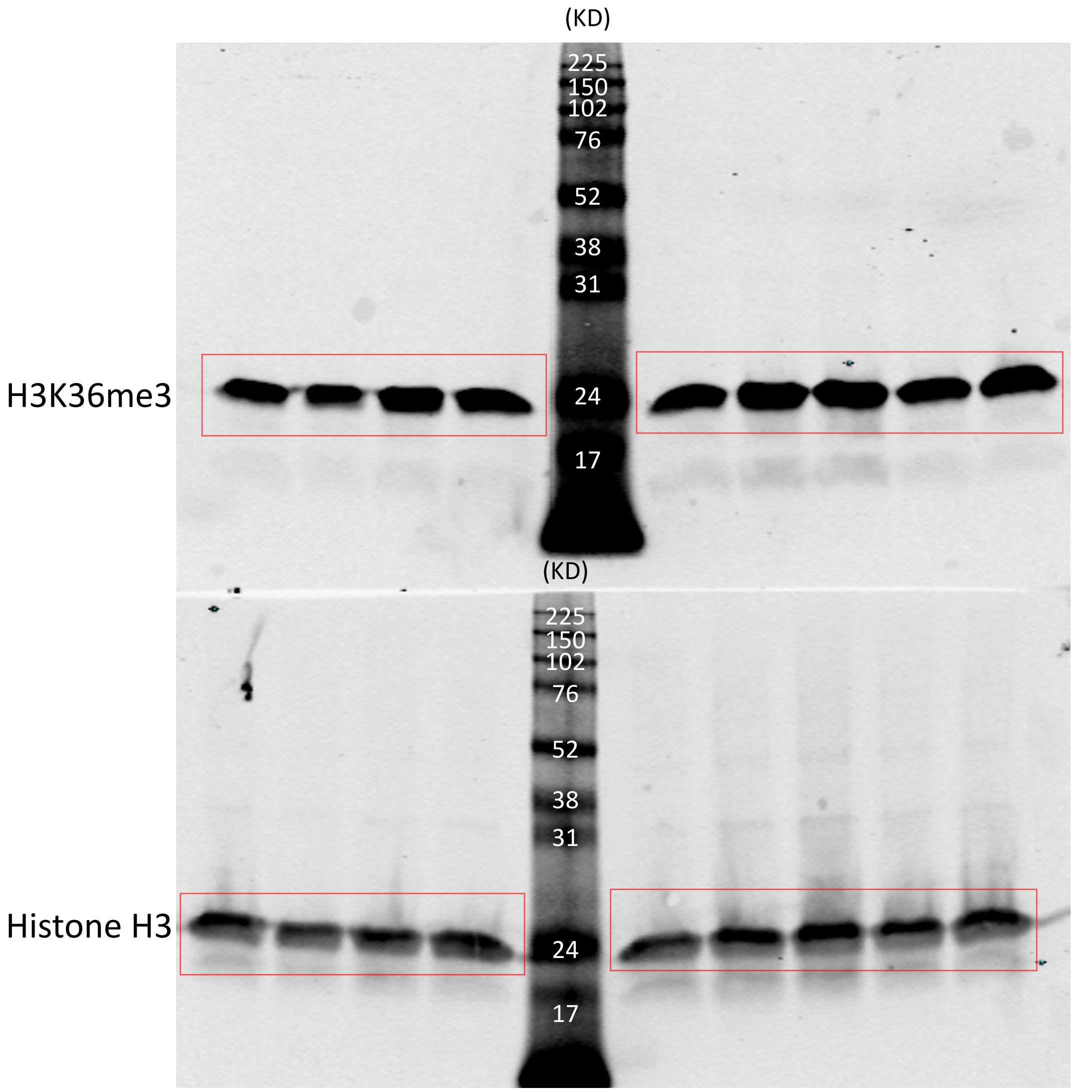

Original images for Figure S6

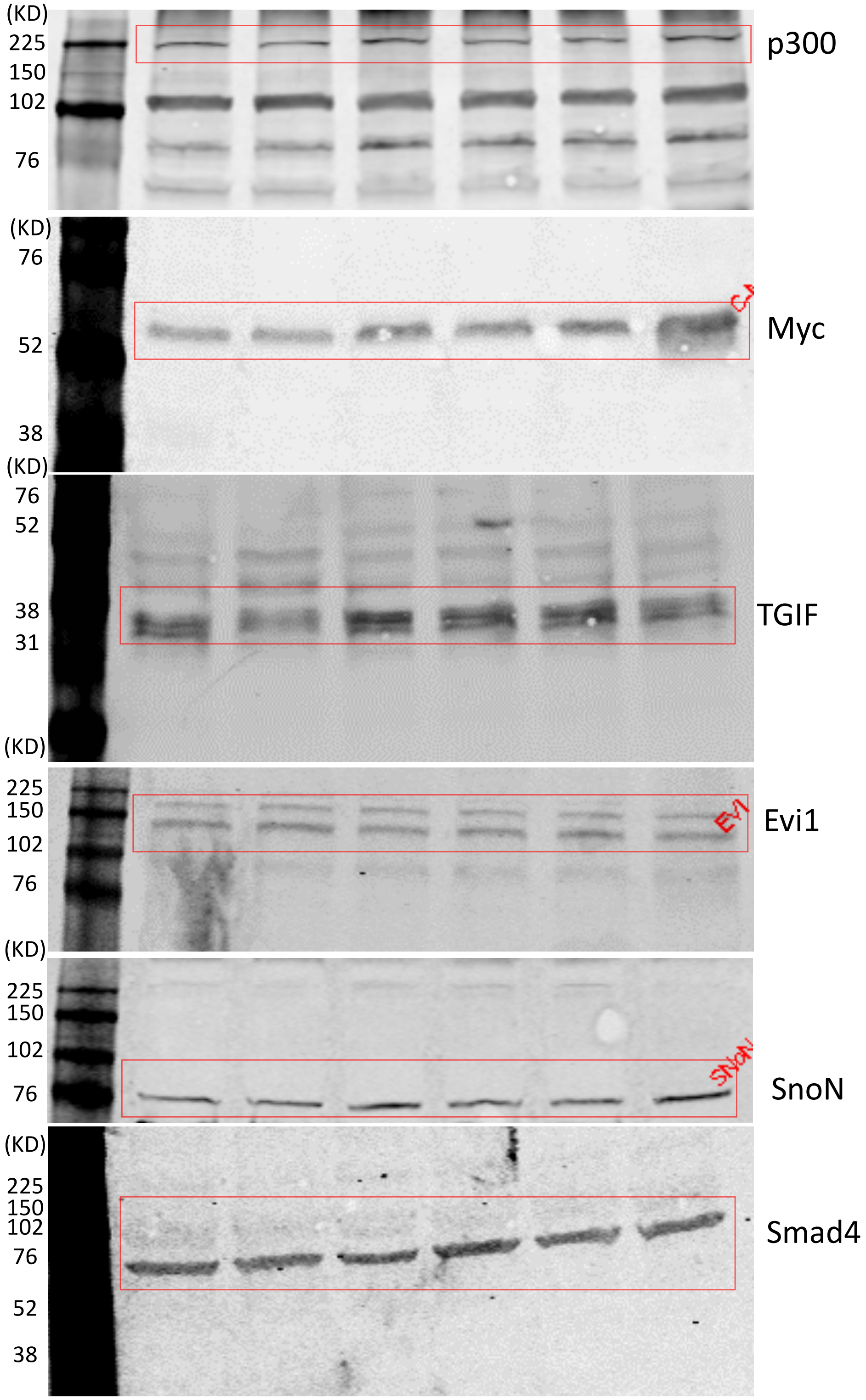

Original images for Figure S4A-continued

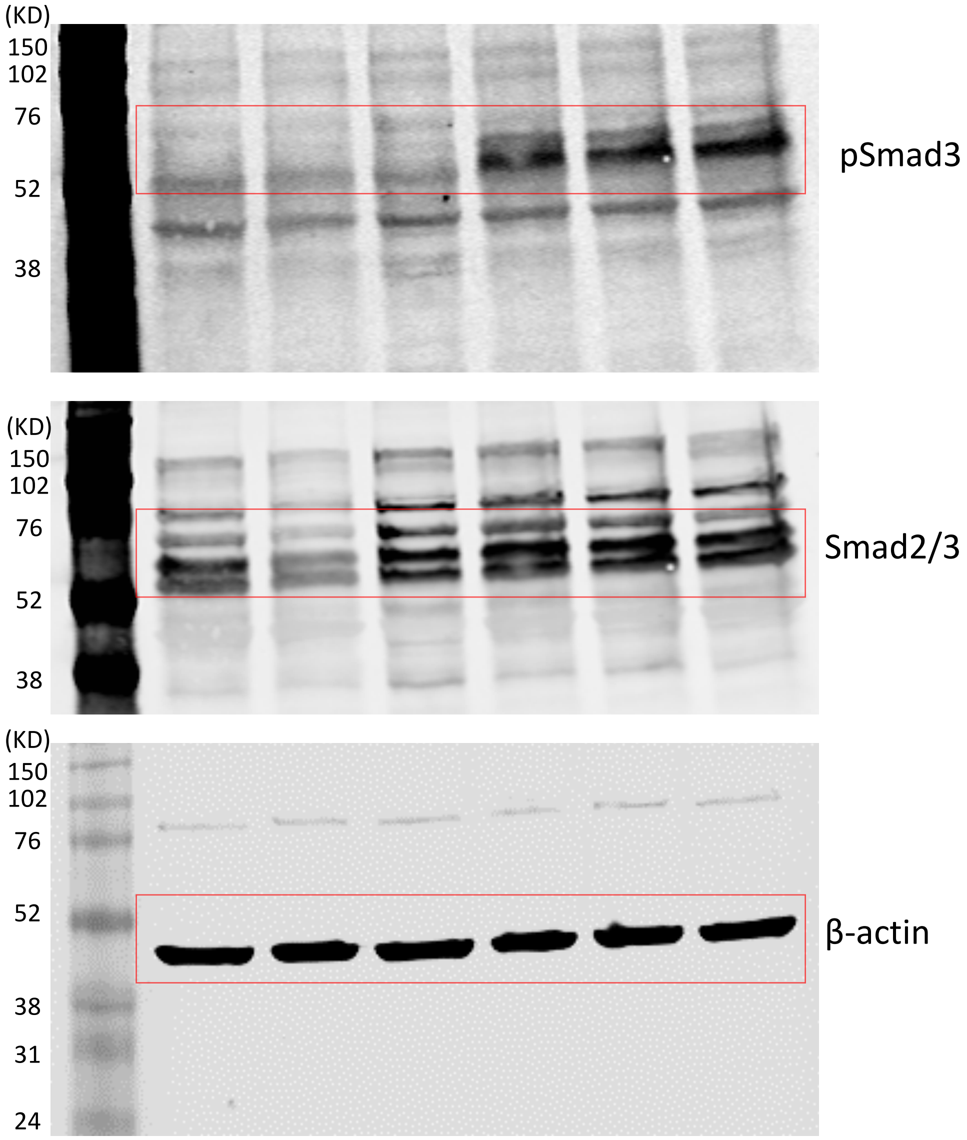

Original images for Figure S7

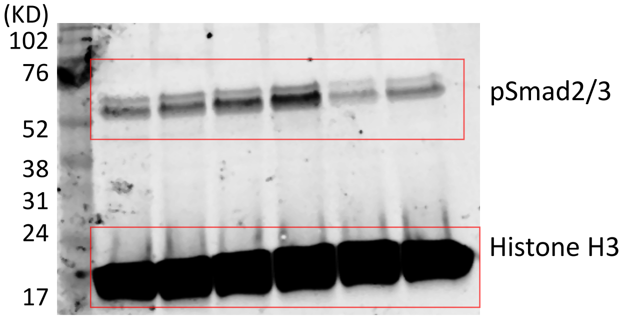

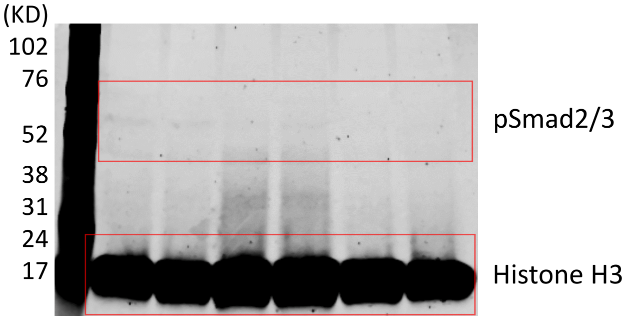

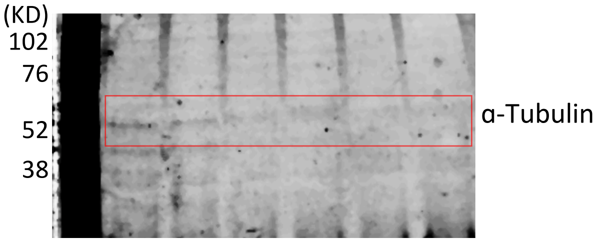

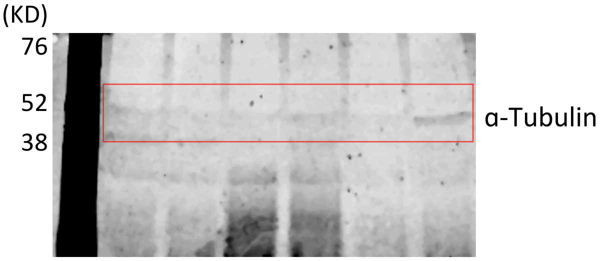

Supplement: S1 Raw Images — (PDF) [file pone.0228160.s003.pdf]
